# Phytochemical Profile, In Vitro Bioactivity Evaluation, In Silico Molecular Docking and ADMET Study of Essential Oils of Three *Vitex* Species Grown in Tarai Region of Uttarakhand

**DOI:** 10.3390/antiox11101911

**Published:** 2022-09-27

**Authors:** Himani Karakoti, Sonu Kumar Mahawer, Monika Tewari, Ravendra Kumar, Om Prakash, Mozaniel Santana de Oliveira, Dharmendra Singh Rawat

**Affiliations:** 1 Department of Chemistry, College of Basic Sciences and Humanities, G.B. Pant University of Agriculture and Technology, Pantnagar 263145, U.S. Nagar, Uttarakhand, India; 2 Campus de Pesquisa-Museu Paraense Emílio Goeldi-Botany Coordination, Av. Perimetral, 1901-Terra Firme, Belém 66077-830, PA, Brazil; 3 Department of Biological Sciences, College of Basic Sciences and Humanities, G.B. Pant University of Agriculture and Technology, Pantnagar 263145, U.S. Nagar, Uttarakhand, India

**Keywords:** natural products, bioactive compounds, antioxidant, phytotoxic, molecular modeling, virtual ligand screening

## Abstract

A comparative study of volatiles, antioxidant activity, phytotoxic activity, as well as in silico molecular docking and ADMET study, was conducted for essential oils from three *Vitex* species, viz., *V. agnus-castus*, *V. negundo*, and *V. trifolia*. Essential oils (OEs) extracted by hydrodistillation were subjected to compositional analysis using GC-MS. A total number of 37, 45, and 43 components were identified in *V. agnus-castus*, *V. negundo*, and *V. trifolia*, respectively. The antioxidant activity of EOs, assessed using different radical-scavenging (DPPH, H_2_O_2_ and NO), reducing power, and metal chelating assays, were found to be significant as compared with those of the standards. The phytotoxic potential of the EOs was performed in the receptor species *Raphanus* *raphanistrum* (wild radish) and the EOs showed different levels of intensity of seed germination inhibition and root and shoot length inhibition. The molecular docking study was conducted to screen the antioxidant and phytotoxic activity of the major and potent compounds against human protein target, peroxiredoxin 5, and 4-hydroxyphenylpyruvate dioxygenase protein (HPPD). Results showed good binding affinities and attributed the strongest inhibitory activity to 13-*epi*-manoyl oxide for both the target proteins.

## 1. Introduction

The plant genus *Vitex* (family: *Verbenaceae*) consists of 250 accepted species and has a wide distribution all over the world, ranging from shrubs to trees in the tropical, sub-tropical regions, and temperate zones [1]. The members of this genus have been widely used in folk medicine and are greatly valued as medicinal plants in several Asian countries, including India, Pakistan, Nepal, China, Sri Lanka, and Bangladesh [2]. The leaves, seeds, flowers, and the whole aerial part of different species of *Vitex* genus have several external and internal uses. The most popular uses of these plants are in the curing of asthma, ophthalmodynia, headaches, coughs, premenopausal syndrome, etc., but various other uses have also been reported [2]. For instance, *V. agnus-castus* fruits are being used in the treatment of menstrual disorders (amenorrhea, dysmenorrhea), and in other female conditions like premenstrual dysphoric disorder, infertility, disrupted lactation, acne, breast pain, menopause, and inflammatory conditions [3,4]. *V. negundo* species is used as tonic, vermifuge, lactagogue, and is also used to treat catarrhal fever, eye diseases, inflammation, skin ulcers, rheumatoid arthritis, and bronchitis [5]. *V. trifolia* is used as a sedative for headaches, as an anti-inflammatory agent, and for the cure of the common cold. The plant is also used for the treatment of cancers in Chinese folk medicine [6,7]. Phytochemical studies revealed that the members of the *Vitex* genus are rich sources of bioactive compounds, including essential oils (terpenoids), flavonoids, glycosides, phenolic acids, ecdysteroids, etc. [7]. Available literature advocated that the essential oils (EOs) and other bioactive compounds from *Vitex* species reported a number of biological activities, such as antioxidant, antibacterial, estrogenic, cytotoxic, antifeedant, antifungal, antidiabetic, enzyme inhibitory, antiproliferative, antipyretic, antimalarial, antinociceptive, and phytotoxic [7,8,9,10,11,12,13].

Modern pharmacological therapies for oxidative stress caused by free radicals are effective, but are costly, and are associated with several undesirable side effects such as carcinogenic and teratogenic effects [14,15]. In respect to the chemical antioxidants, the search for alternative sources, based on natural, plant-based origins, is increasing nowadays, as they are supposed to be safer. Essential oils from a wide number of plant sources have been tested and have been reported to have excellent antioxidant properties [16,17,18,19]. Similarly, in recent years, research regarding the pesticidal properties of plant-based products (botanicals) has been gradually increasing as they are safer to the environment, easily degradable, and have low toxicity as compared to the chemical pesticides [20]. Moreover, in previous studies, the essential oil obtained from *V. negundo* and *V. agnus-castus* has been reported to cause potent phytotoxic activity [21,22], however, there is no report on the phytotoxic potential of *V. trifolia*. EOs are excellent options among natural sources, as they are sources of highly phytotoxic allelochemicals that affect the growth and development of plants and unwanted plants (weeds) [23].

Studies highlighted *Vitex* species as a source of antioxidant and phytotoxic agent due to their essential oil composition [7,21,22]. The efficiency and activity of the essential oil greatly depends on its chemical constituents, which depends on the genotypes of the plant and on the environmental, climatic, and agronomic conditions [24]. The essential oils of *V. agnus castus* and *V. trifolia*, from the Tarai region of Uttarakhand, have not been screened for their phytochemical analysis. Therefore, briefly, the present study aims (i) to check the chemical diversity among the essential oil composition of *Vitex agnus-castus*, *Vitex negundo*, and *Vitex trifolia* (from Tarai region of Uttarakhand, India); (ii) to evaluate the in vitro antioxidant and phytotoxic (herbicidal) activities; (iii) to carry out the in silico studies about the inhibitory effect of major volatiles of the essential oils on the crystal structures of some proteins; (iv) to perform the ADMET prediction of major compounds identified in essential oils under investigation.

## 2. Materials and Methods

### 2.1. Collection of Plant Material

The fresh leaves of different plant species of the *Vitex* were collected from Pantnagar (28°58′12″ N, 79°24′36″ E), Tarai region of Uttarakhand, India. The plant specimens were identified by one of the authors (D.S. Rawat), a taxonomist. The voucher specimen for different identified *Vitex* species viz., *Vitex agnus-castus*, *Vitex negundo* L., and *Vitex trifolia* L., with voucher numbers GBPUH-1439, GBPUH-1438, and GBPUH-1440, were deposited at the herbarium of Department of Biological Sciences, for future references.

### 2.2. Extraction of Essential Oil

Fresh leaves of different *Vitex* species were subjected to hydrodistillation for 4 h to isolate essential oils using a Clevenger-type apparatus, and the isolated essential oils were designated as VAO, VNO, and VTO for *Vitex agnus-castus*, *Vitex negundo*, and *Vitex trifolia*, respectively. The obtained essential oils were dried over anhydrous sodium sulphate (Na_2_SO_4_) in order to remove any trace of water and then stored in amber color glass vials at a low temperature (4 °C in refrigerator) for further uses. The oil yield (*v*/*w*) was recorded as 0.9% (0.45 mL/100 gm dry matter), 0.8% (0.4 mL/100 gm dry matter), and 0.6% (0.3 mL/100 gm dry matter) for VAO, VNO, and VTO, respectively.

### 2.3. Chemical Composition Analysis

To check the chemical diversity in tested *Vitex* species, the essential oils were analyzed by GC-MS (Shimadzu QP 2010 plus) with GCMS-QP 2010 Ultra DB-5 and GCMS-QP 2010 Ultra Rtx-5MS column (30 m × 0.25 mm i.d., 0.25 µm). The following experimental conditions used helium as the carrier gas (flow rate = 1.21 mL/min, split ratio = 10.0). Oven temperature was programmed at 50–280 °C with a temperature gradient of 3 °C/min up to 210 °C (isotherm for 2 min), then 6 °C/min up to 280 °C. Identification of essential oil components was done by comparing their relative retention index (RI) values with mass spectra NIST (NIST version 2.1) and WILEY (7th edition) libraries, and by matching the fragmentation pattern of the mass spectral data with those reported in the literature [25,26]. 

### 2.4. Antioxidant Activity

Different in vitro tests were performed to evaluate the antioxidant activity of the essential oils, and the results were presented as mean ± SD of triplicate.

#### 2.4.1. DPPH Radical Scavenging Assay

Previously proposed methods have been followed to perform the assay [27,28]. In brief, different concentrations of VAO, VNO, and VTO (10 μL/mL–50 μL/mL) were added to 5 mL of freshly prepared methanolic solution of DPPH (0.004%), solution was kept for incubation under dark for half an hour, and further, the absorbance was taken in triplicates at 517 nm in a UV spectrophotometer (Thermo Fisher Scientific, Evolution-201, Waltham, MA, USA) against a blank. The standard antioxidant used was BHT, in the same concentrations as the tested essential oils (10 μL/mL–50 μL/mL). The % inhibition of DPPH free radical of the oils and standard was calculated by using the following formula:

$$\%\;\mathrm{DPPH}\;\mathrm{radical}\;\mathrm{scavenging}\;\mathrm{activity}=\frac{\left(A_{o}\;–{\;A}_{t}\right)}{A_{o}}\times 100$$

where A_o_ and A_t_ are the absorbance values of control and test essential oils, respectively. Percent inhibition was plotted against concentrations, and the equation for the line was used to obtain the IC_50_ (half-maximal inhibitory concentration) values.

#### 2.4.2. Hydrogen Peroxide (H_2_O_2_) Radical Scavenging Activity

H_2_O_2_ radical scavenging activity of tested samples was performed as per the prescribed protocol reported earlier [29,30]. Here, 0.6 mL of H_2_O_2_ solution (40 mM) prepared in phosphate buffer (0.1 M; pH 7.4) was added to 0.4 mL methanolic solution of different concentrations of essential oils and the standard (10–50 µL/mL). The above solution was incubated at room temperature for 10 min. Further, the absorbance was taken at 230 nm against the blank, i.e., methanol. Here, *L*-ascorbic acid (10–50 µL/mL) was taken as a positive control. The percentage scavenging of H_2_O_2_ was calculated by using the following formula:

$$\%\;H_{2}O_{2}\;\mathrm{radical}\;\mathrm{scavenging}\;\mathrm{activity}=\frac{\left(A_{o}-{\;A}_{t}\right)}{A_{o}}\times 100$$

where A_o_ and A_t_ are the absorbance values of control and test essential oils, respectively. Percent inhibition was plotted against concentrations and the equation for the line was used to obtain the IC_50_ values.

#### 2.4.3. Nitric Oxide Radical Scavenging Activity

The nitric oxide (NO) radical scavenging activity of the tested essential oils was determined by the method as described earlier [31]. Briefly, 2 mL of sodium nitroprusside (10 mM) prepared in phosphate buffer saline (0.5 mM, pH 7.4) was added to different concentrations of essential oils and the standard (10–50 µL/mL) separately, and incubated at 25 °C for 150 min. Further, 0.5 mL of griess reagent containing 1.0 mL sulphanilic acid reagent was added to 0.5 mL of each incubated solution. The mixture was again incubated for 30 min at room temperature and the absorbance was taken at 540 nm. *L*-ascorbic acid was taken as the standard antioxidant. The percentage scavenging of NO was calculated by using the following formula:

$$\%\;\mathrm{NO}\;\mathrm{radical}\;\mathrm{scavenging}\;\mathrm{activity}=\frac{\left(A_{o}\;–{\;A}_{t}\right)}{A_{o}}\times 100$$

where A_o_ and A_t_ are the absorbance values of control and test essential oils, respectively. Percent inhibition was plotted against concentrations and the equation for the line was used to obtain the IC_50_ values.

#### 2.4.4. Reducing Power Assay

The reducing power assay of different essential oils was determined by the method developed earlier [32]. Different concentrations of tested samples (essential oils and the standard (10–50 μL/mL)) were added to 2.5 mL of phosphate buffer (200 mM, pH = 6.6). Further, 2.5 mL of 1% potassium ferricyanide, K_3_[FeCN_6_], was added to the above solution. The solution was incubated for 20 min at 50 °C and then 2.5 mL of trichloroacetic acid was added to the incubated solution, followed by centrifugation at 650 rpm for 10 min. 5 mL of distilled water and 1 mL of 0.1% ferric chloride were added to the upper layer (1 mL). The absorbance of the final solution was taken at 700 nm, and gallic acid (10–50 µL/mL) was taken as a positive control. The percentage reducing power was calculated by using the following formula:

$$\%\;\mathrm{Reducing}\;\mathrm{power}\;\mathrm{activity}=\frac{\left(A_{o}\;–{\;A}_{t}\right)}{A_{o}}\times 100$$

where A_o_ and A_t_ are the absorbance values of control and test essential oils, respectively. RP_50_ values were calculated using regression equations for the percent inhibition plotted against concentrations. 

#### 2.4.5. Fe^2+^ Metal Chelating Activity

The Fe^2+^ metal-chelation activity of VAO, VNO, and VTO was measured as per the prescribed and developed protocol [33]. Different concentration of oils (10–50 µL/mL), as well as the standard, were mixed with 0.1 mL of FeCl_2_·4H_2_O (2 mM) and 0.2 mL of (5 Mm) ferrozine separately. Further, methanol (4.7 mL) was added to the solution, making the final volume 5 mL. The solution was shaken and was incubated for 30 min at 25 °C, and the absorbance was taken at 562 nm using spectrophotometer (Thermo Fisher Scientific, Evolution-201, USA). Na_2_-EDTA (10–50 µL/mL) was used as a standard antioxidant. The ability of the samples to chelate ferrous ion was calculated using the following formula:

$$\%\;{\mathrm{Fe}}^{2+}\;\mathrm{metal}\text{-}\mathrm{chelation}\;\mathrm{activity}=\frac{\left(A_{o}\;–{\;A}_{t}\right)}{A_{o}}\times 100$$

where A_o_ and A_t_ are the absorbance values of control and test essential oils, respectively. IC_50_ values were obtained using regression equations for the plots of percent inhibition against concentrations.

### 2.5. Herbicidal (Phytotoxic) Activity

The herbicidal activity on the receptor plant, *Raphanus raphanistrum*, was carried out with essential oils of *Vitex* species. Different parameters were used, such as inhibition of seed germination, inhibition of shoot, and root length growth, using the method reported earlier [34,35]. For the experiment, radish seeds were obtained from the VRC (Vegetable Research Centre), G.B.P.U.A. & T. Pantnagar, Uttarakhand, India. 

#### 2.5.1. Seed Germination Inhibition

For evaluating the seed germination inhibition, different concentrations of essential oils (50–200 µL/mL) were prepared in Tween-20 (1%) solution of distilled water. In order to break dormancy, radish seeds were surface sterilized in 5% hypochlorite solution for 15 min. Ten sterilized seeds of radish were placed in each petri plates, which were lined with sheets of qualitative filter papers. Further, 2 mL of various concentrations of the tested sample (50–200 µL/mL) were applied onto the plates and the seeds were allowed to germinate at controlled condition of 25 ± 1 °C and a photoperiod of 12 h in an incubator. Seeds with a root length of 2 mm were considered germinated. Distilled water was taken as the control while pendimethalin (50–200 µL/mL) was used as a standard herbicide, and the bioassay was performed in triplicate. After 120 h, the numbers of germinated seeds in each petri dish were counted, followed by the calculation of percent seed germination inhibition values using the following formula:

Inhibition of seed germination (% Inhibition) = 100 × (1 − Gt/Gc)

where Gt = no. of seeds germinates in treatment, Gc = No. of seeds germinate in control.

#### 2.5.2. Inhibition of Shoot and Root Elongation

Assessment of shoot and root elongation were performed at controlled condition of 25 °C for a photoperiod of 24 h. Each Petri dish received 2.0 mL of the test solution, and two pre-germinated seeds were placed in each petri plate. The EOs were tested at the same concentrations as the germination bioassay. At the end of the 120 h of incubation, the length of the shoot and root were measured. Distilled water was taken as the controlled treatment while pendimethalin (50–200 µL/mL) was used as a standard herbicide, and the bioassays were performed in triplicate. The formulae used for determining the inhibition of shoot and root growth were as follows:

Inhibition of hypocotyl (shoot length) growth (% Inhibition) = 100 × (1 − Ct/Cc)

where, Ct = shoot length growth in treatment, Cc = shoot length growth in control.

Inhibition of radicle (root length) growth (% Inhibition) = 100 × (1 − Rt/Rc)

where, Rt = root length growth in treatment, Rc = root length growth in control.

### 2.6. Molecular Docking Studies

Virtual ligand screening is an in silico method used to dock small molecules (ligand) to macromolecule (protein) to discover potent compounds that have the necessary biological effect [36]. The molecular docking study of the selected volatiles from VAO, VNO, and VTO was carried out on 4-hydroxyphenylpyruvate dioxygenase (HPPD) receptors, as this protein has been reported as a molecular target for compounds with post-emergence herbicidal activity [37,38], and the second protein taken was human peroxiredoxin 5, which has a broader activity against reactive oxygen species [39]. The three-dimensional (3D) structures of the HPPD and human peroxiredoxin 5 proteins were obtained from the RCSB ProteinData Bank with PDB ID: 6J63 and 1HD2, respectively. The 3D structures of the selected proteins converted into PDB formats by deleting the water molecules, HETATOMS, and adding polar hydrogens using Biovia Discovery Studio-2021 Client. The compounds from the essential oils for docking studies were selected based on their higher percentage contents and their concerned structures were obtained from the PUBCHEM database (https://pubchem.ncbi.nlm.nih.gov/, accessed on 12 August 2022) in the SDF (structure data file) format. The selected compounds were 1,8-cineole (CID:2758), sabinene (CID:18818), α-pinene (CID:6654), α-terpinyl acetate (CID:111037), β-farnesene (CID:5281517), viridiflorol (CID:11996452), β-caryophyllene (CID:5281515), β-iraldiene (CID: 5375218), terpine-4-ol (CID:2724161), 5-(1-isopropenyl-4,5-dimethylbicyclo[4.3.0]nonan-5-yl)-3-methyl-2-pentenyl acetate (CID:5375240), 13-*epi*-manoyl acetate (CID:18529657), α-phellandrene (CID:7460), and caryophyllene oxide (CID:1742210). Structures of the ligands in their SDF format were then imported into PyRx Software using an open babel tool embedded in PyRx software. Energy minimization (optimization) was performed by adding charges and optimizing the universal force field. Further, the ligands were converted into AutoDock Ligand format (PDBQT). To find out the binding affinity and to know the various ligand–receptor interactions responsible for the antioxidant and phytotoxic activity, the molecular docking of the selected major constituents was performed using PyRx with Vina Wizard tool. The protein and multiple ligands to be docked were selected in the PyRx software using the Vina Wizard Control. The “Run Vina” control was selected to initiate the docking process. The results were observed by selecting the “Analyze Vina” tool and exported as CSV files [36]. Biovia Discovery Studio-2021 Client was used for the visualization of 2D and 3D interactions of docking poses.

### 2.7. In Silico ADMET Study

The structures of the selected compounds from the essential oils were drawn using ChemDraw Ultra 8.0 for the pharmacokinetics (absorption, distribution, metabolism, and excretion (ADME)) studies. The legends were converted into the SMILES format and then the drug-like and pharmacokinetic properties of the selected compounds were predicted using ADME tool by a SwissADME online server (http://www.swissadme.ch/, accessed on 12 August 2022), as per the developed protocol [40]. ProTox-II webserver (http://tox.charite.de/protox_II, accessed on 12 August 2022) was used to study the toxicity profile. It calculates the prediction based on different parameters such as organ toxicity (hepatotoxicity), oral toxicity, and toxicological endpoints (cytotoxicity, mutagenicity, carcinotoxicity, and immunotoxicity).

### 2.8. Statistical Analysis

Two and one factor Analysis of variance (ANOVA), followed by the Tukey test, was performed using RStudio (Version 2021.09.2) developed by RStudio team, PBC, Boston, MA and OriginPro, Version 2022b developed by OriginLab, Northampton, MA, USA Student trial version software, respectively, to analyze the significant difference among the treatment means. The *p* value < 0.05 was considered to show the significant difference. All the data in the experiment were reported as mean ± SD (standard deviation). To define the variability in different essential oils based on chemical composition, Chemometric Analysis was performed based on the heatmap clustering using heatmapper, free web server available (http://www.heatmapper.ca, accessed on 12 August 2022) developed at University of Alberta, Canada [41]. We performed Principal Component Analysis (PCA) on chemical composition for the three *Vitex* species under investigation to identify the most significant features in the dataset and Pearson’s correlation test to analyze the correlation among the chemical compounds of essential oils, and their biological activities were performed using OriginPro, Version 2022b.

## 3. Results and Discussion

### 3.1. Chemical Composition

A total of 37, 45, and 43 components were detected in VAO (0.1–25.0%), VNO (0.1–19.4%), and VTO (0.1–16.2%), respectively. A total of 22 components were found to be common in all three EOs, which were as follows: α-thujene, α-pinene, sabinene, β-pinene, myrcene, 1,8-cineole, γ-terpinene, *p*-cymene, linalool, *trans*-sabinenehydrate, *cis*-*p*-menth-2-en-1-ol, terpinen-4-ol, α-terpineol, dihydroedulan II, β-caryophyllene, α-humulene, β-iraldeine, β-caryophyllene oxide, α-muurolol, drimenol, and manool. However, they varied in their relative percentage. As summarized in Table 1, 1,8-Cineole (25.0%), sabinene (13.3%), α-pinene (8.2%), and α-terpinyl acetate (5.5%) were the dominant compounds in *V. agnus-castus* oil; sabinene (19.4%), viridiflorol (17.8%), β-caryophyllene (7.5%), and β-iraldiene (6.4%) were dominant in *V. negundo* oil, while β-caryophyllene (16.2%), 5-(1-isopropenyl-4,5-dimethylbicyclo[4.3.0]nonan-5-yl)-3-methyl-2-pentenol acetate (11.7%), 13-epi-manoyl oxide (5.6%), and caryophyllene oxide (4.6%) were the abundant compounds in *V. trifolia* oil. In terms of chemical class composition, VAO was dominated by oxygenated monoterpene (40.6%), followed by monoterpene hydrocarbons (31.2%) and others. The only diterpenoid present in VAO was manool (0.5%). On the other hand, VNO was mainly dominated by monoterpene hydrocarbons (29.4%), followed by oxygenated sesquiterpenes (24.8%) and oxygenated monoterpenoids (11.3%). The most abundant class found in VTO was sesquiterpene hydrocarbon (21.9%) followed by oxygenated sesquiterpene (15.8%) and oxygenated diterpenes (13.8%). For more details on chromatograms, and chemical composition mass spectra, please refer to Supplemental Material S1.

Rezaei et al. [42] evaluated the effects of different irrigation regimes on the essential oil composition of *V. agnus-castus* under three shading levels, collected from Isfahan, Iran, and reported α-pinene (16.4–60.7%), β-terpinyl acetate (15.4–40.2%), caryophyllene (2.7–9.0%), and camphene (0.09–6%) as the main constituents. The compounds 1,8-cineole, sabinene, myrcene, α-thujene. α-terpineol, β-farnesene, spathulenol, β-caryophyllene oxide, humulane-1,6-dien-3-ol, and manool were not detected in any of the samples studied in this work, although these are present in noticeable amounts in the current study. However, in other studies, 1,8-cineole and sabinene were reported as the main EO constituents of *V. agnus castus* leaves [43,44,45]. These findings are generally consistent with those of the current investigation. The oxygenated sesquiterpenes identified in VAO, such as spathulenol, ledol, and *epi*-α-cadinol, have previously been identified in the EO of *V. agnus castus* leaves collected in Ogliastra, Sardinia, Italy [46], however, other sesquiterpenoids such as β-eudesmol, drimenol, and flourensadiol were not detected previously in *V. agnus castus* leaves essential oil. VAO also lacks compounds like limonene, viridiflorol, and globulol that are often present in most of the previous reports [45,46,47,48]. Thus, the study demonstrates different chemo-variants of *V. agnus castus* both qualitatively and quantitatively.

Previous researchers have also studied the EOs of *Vitex negundo* under investigation herein. For instance, the major compounds detected in VNO, sabinene (19.4%), viridiflorol (17.8%) and β-caryophyllene (7.5%), were also found to be present in the hydrodistilled *Vitex negundo* leaves EO in variable amounts [22,49,50]. 5-(1-Isopropenyl-4,5-dimethylbicyclo[4.3.0]nonan-5-yl)-3-methyl-2-pentenol acetate (5.2%), another major compound detected in VNO, was also found to be present in leaves essential oil of *V. negundo* in notable amounts [51]. The chemical composition of EO of *Vitex negundo* extracted during the spring season from the same location (Pantnagar) revealed the presence of over 33 compounds, in which the major compounds detected were viridiflorol (23.8%), sabinene (11.2%), unidentified diterpene M^+^ = 272 (11.0%), and caryophyllene (6.7%) [50]. The composition was lacking the compounds, α-thujene, β-pinene, α-terpinene, β-phellandrene, γ-terpinene, linalool oxide, *p*-cymene, *trans*-sabinenehydrate, theaspirane A, β-iraldeine, β-caryophyllene oxide, ledol, humulane-1,6-dien-3-ol, and the diterpenes cubetene, phytol, manool, and sclereol, however, in the present study, these compounds are detected in noticeable amounts. Thus, the composition might vary as a result of the harvesting season. However, in another study of Indian origin, α-copaene (25.3%), β-elemene (19.2%), and camphene (21.1%) were reported as the predominant compounds in leaf essential oil of *V. negundo* [52]. Khokra et al. [53] reported ethyl-9-hexadecenoate (28.5%), δ-guaiene (18.0%), and caryophyllene oxide (10.2%) as the major components in leaf essential oil of *V. negundo.* On the other hand, the leaf essential oil of *V. negundo* from Chinese origin revealed δ-guaiene (50.0%) and β-caryophyllene (38.0%) as the major constituents [54]. Both qualitative and quantitative variations in essential oils of *V. negundo* from different geographic regions might be due to the different geographical and climatic conditions.

Thomas et al. [55], investigated the essential oil of *V. trifolia* and obtained caryophyllene (38.36%) and 1,8-cineole (25.72%) as the predominant compounds. However, in the present study, the amount of 1,8-cineole is only 2.1% in VTO. β-caryophyllene is also identified as the major constituent of *V. trifolia* oil by several other reports [56,57,58], which is in agreement with the present study. Arpiwi et al. [59] detected five components in *V. trifolia* essential oil in which *cis*-ocimene (44.57%), α-thujene (25.63%) and cyclopentene,3-isopropenyl-5,5-dimethyl (18.19%) were identified as the major constituents. However, in the present study such compounds were not detected, and the amount detected for α-thujene was also negligible (0.2%). The second major compound detected in VTO, 5-(1-isopropenyl-4,5-dimethylbicyclo[4.3.0]nonan-5-yl)-3-methyl-2-pentenolacetate (11.7%), has also been found in other *Vitex* species such as *V. agnus castus* and *V. negundo* [51,60]. The noticeable diterpenes identified in VTO, 13-epi-manoyl oxide (5.6%), and 16-oxo-cleroda-3,13(14)-(e)-dien-15-oic acid (2.8%) are also being detected for the first time in *V. trifolia* oil. These differences in the essential oil constituents might be due to internal and external factors and their interactions.

In addition, the compounds identified in the tested essential oils have potent biological applications. 1,8-cineole is used in cosmetic products and as a flavoring agent because of its pleasant aroma and taste. The compound has several other properties: insecticidal, antioxidant, and anti-inflammatory [61]. Viridiflorol has prominent use as an anti-inflammatory, antioxidant, and anti-tuberculosis agent [62]. Sabinene has antimicrobial, anti-inflammatory, and antioxidant properties described in literature [63]. Further, the diterpene, 13-*epi*-manoyl oxide, has cytotoxic antibacterial and antifungal activities [64].

### 3.2. Chemometric Analysis

**Figure 1:**
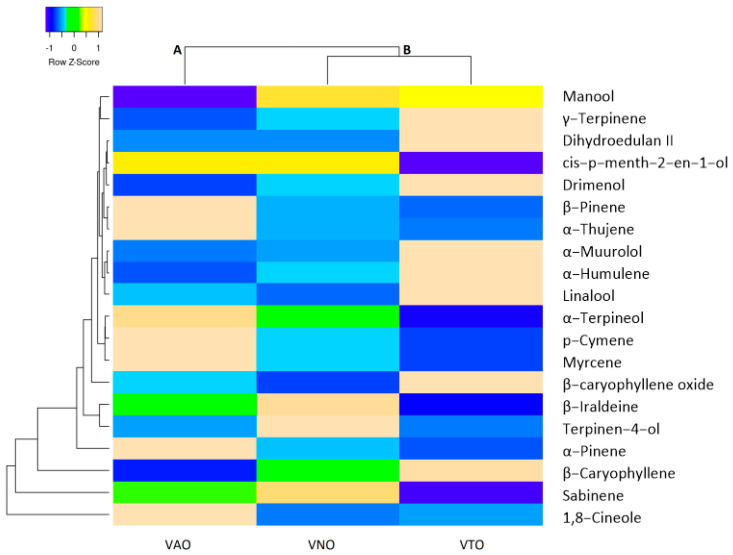
The heatmap analysis of the common essential oil constituents and tested species (The distribution of trait (common essential oil components) was identified by colors, where yellow color showed the maximum value of the trait, and blue color represented the minimum value).

The main chemical components common for all the essential oils of tested species (α-thujene, α-pinene, sabinene, β-pinene, myrcene, 1,8-cineole, γ-terpinene, *p*-cymene, linalool, cis-p-menth-2-en-1-ol, terpinen-4-ol, α-terpineol, dihydroedulan II, β-caryophyllene, α-humulene, β-iraldeine, β-caryophyllene oxide, α-muurolol, drimenol, and manool) were compared with hierarchical cluster analysis with Euclidean distance as the similarity index. The heat map clustering diagram is depicted in Figure 1. 1,8-Cineole, sabinene, and β-caryophyllene form separate clusters with different values compared to the rest of the analyzed common constituents. Based on Euclidean distance in the heat map clustering, all the tested species are clearly divided into two main clusters on the basis of their common chemical constituents. VNO and VTO are clustered in one cluster, whereas VAO is in the separate cluster.

### 3.3. Principal Component Analysis

**Figure 2:**
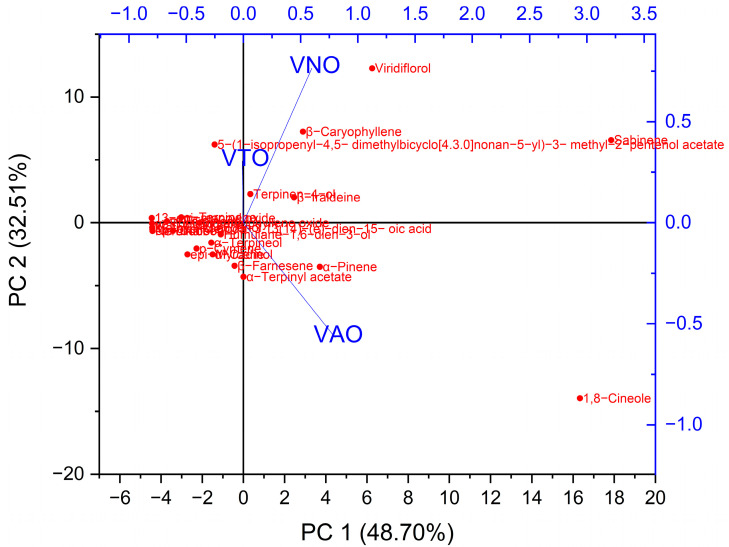
Principal Component Analysis of tested essential oil’s chemical constituents.

Principal component analysis (PCA) is one of the greatest multivariate statistical techniques used to identify a dataset’s most important features. To assess the chemical profiling changes caused by interspecies as well as altitudinal influences, distinct essential oils can be used in PCA pattern recognition. The PCA approach determined that the cumulative contribution rate of variance of the first two principal components (PC1 and PC2) could account for 81.2% of the variance information for changes in chemical composition. In order to define the compositional variations in the essential oils, PC1 and PC2 were used. PC1 was favorably linked with terpinen-4-ol, β-iraldeine, β-caryophyllene, viridiflorol, and sabinene, and contributed 48.7% of the total variance. However, PC2 makes up 32.5% of the variation and has a strong positive correlation with α-pinene and 1,8-cineole (Figure 2).

### 3.4. Antioxidant Activity

**Figure 3:**
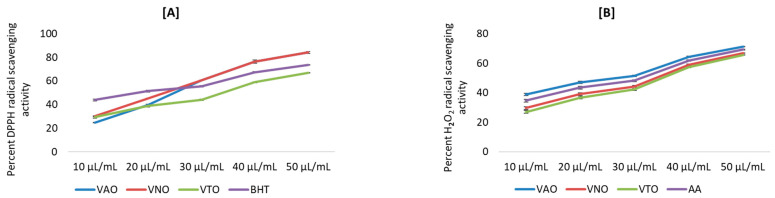

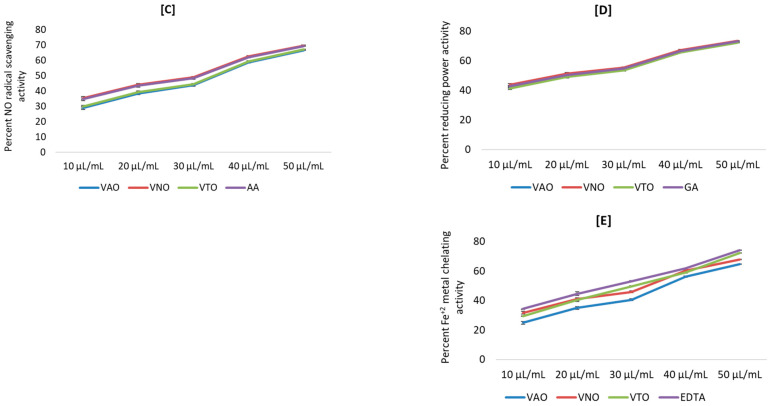
(**A**–**E**) Antioxidant activity for essential oils of *Vitex* species; (**A**) Percent DPPH radical scavenging activity; (**B**) Percent H_2_O_2_ scavenging activity; (**C**) Percent NO radical scavenging activity; (**D**) Percent reducing power activity; (**E**) Percent Fe^2+^ metal chelating activity.

The antioxidant activity was determined by using different chemical-based methodologies. Figure 3A–E depict the antioxidant activity of tested essential oil in terms of percent inhibition. Results revealed that all the antioxidant activities were in a concentration-dependent manner. The percent inhibition of free radicals (DPPH, H_2_O_2_, NO), reducing power, and metal chelation increased, with increasing concentration from 10 µL/mL to 50 µL/mL. Further, the percent inhibition by the tested essential oils and the standards for different antioxidant assays were plotted against concentrations, and the equation for the line was used to obtain the IC_50_ (half-maximal inhibitory concentration) values.

**Figure 4:**
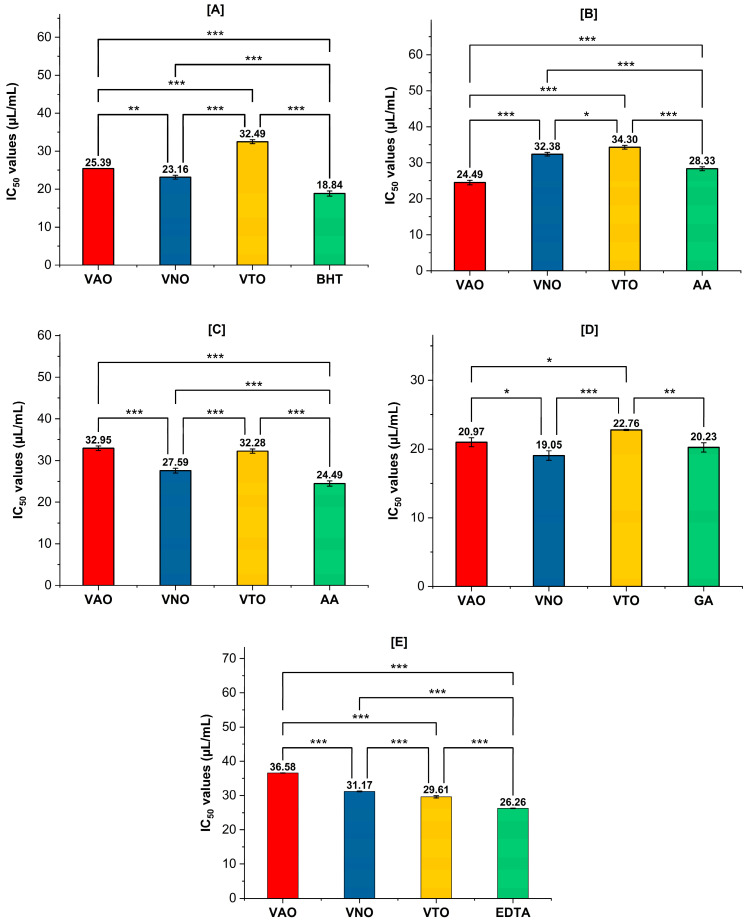
(**A**–**E**) Antioxidant activity in terms of IC_50_ values (µL/mL) for VAO, VNO, and VTO, (**A**) DPPH radical scavenging, (**B**) H_2_O_2_ radical scavenging, (**C**) NO radical scavenging, (**D**) reducing power activity, (**E**) Metal chelating activity. Statistically significant differences were examined using one-way ANOVA and Tukey posthoc tests. *** *p <* 0.001, ** *p <* 0.005, * *p <* 0.05 above columns indicate significant differences between treated groups. Values are mean ± SD, *n* = 3.

Figure 4A–E represent the antioxidant activity of tested essential oils in terms of their IC_50_ values. In the DPPH assay, the reduction of the stable radical DPPH (violet) to the yellow-colored DPPH-H is employed to measure the potential of an antioxidant molecule to act as a donor of hydrogen atoms or electrons. Figure 4A shows that VNO reduced DPPH with an IC_50_ value of 23.16 ± 0.5 µL/mL, which is close to the standard antioxidant taken for the assay, BHT (18.84 ± 0.6 µL/mL). VAO and VTO displayed moderate and weak antioxidant activity, with IC_50_ 25.39 ± 0.0 µL/mL and 32.49 ± 0.5 µL/mL, respectively. H_2_O_2_ can cross the biological membrane, and as a result it can damage the human body by forming reactive OH· radicals following Fenton reaction [65]. In H_2_O_2_ radical scavenging assay, VAO (IC_50_ = 24.49 ± 0.1 µL/mL) displayed good scavenging activity when compared to the standard, ascorbic acid (28.33 ± 0.5 µL/mL), followed by VNO (32.38 ± 0.5 µL/mL) and VTO (34.30 ± 0.5 µL/mL). The extent of nitrite scavenging by the samples was compared with ascorbic acid and showed IC_50_ values as: ascorbic acid (24.49 ± 0.1 µL/mL) > VNO (27.58 ± 0.1 µL/mL) > VTO (32.27 ± 0.1 µL/mL) > VAO (32.95 ± 0.5 µL/mL). The reducing power of a compound is related to its ability to transfer electrons, which indicates its significant antioxidant potential. As shown in Figure 4D, VNO displayed good reducing capability (RP_50_ = 19.05 ± 0.6 μL/mL) that is very close and lower than that of the standard gallic acid (20.22 ± 0.4 μL/mL). The order of RP_50_ values for different samples is in the order: VNO (19.05 ± 0.6 μL/mL) > gallic acid (20.22 ± 0.4 μL/mL) > VAO (20.97 ± 0.5 μL/mL) > VTO (22.74 ± 0.7 μL/mL). In auto-oxidation reactions, metal ion is a powerful catalyst as it can inhibit the generation of oxygen radicals. The IC_50_ values of different samples and standards towards their antioxidant potentiality in terms of chelating ability were observed as: Na_2_-EDTA (IC_50_ = 26.23 ± 0.26 µL/mL) > VTO (IC_50_ = 29.77 ± 0.2 µL/mL) > VNO (IC_50_ = 31.18 ± 0.2 µL/mL) > VAO (IC_50_ = 36.60 ± 0.1 µL/mL).

Such high antioxidant activity of VNO for the DPPH and NO radical scavenging is likely due to high amount of sabinene as well as other constituents of VNO such as β-caryophyllene, terpinen-4-ol, 1,8-cineole, which already possess antioxidant potential via different parameters [66,67,68]. Additionally, Kazemi [69] showed that sabinene exhibited potent NO-scavenging effect and inhibited the expression of inducible NO synthase. Similar results were observed in previous studies in antioxidant activity of *V. negundo* essential oil in which the major component was sabinene [49]. In H_2_O_2_ radical scavenging assay, VAO showed good scavenging activity, which may be due to the presence of 1,8-cineole, sabinene, and β-caryophyllene as the major constituents [69,70]. In earlier reports, essential oil and extracts of aerial parts of *V. agnus castus* have been tested for antioxidant activity as having a high amount of 1,8-cineole and β-caryophyllene in their composition, and the samples showed good antioxidant activity [64,71,72]. Since essential oils are complexed mixtures of number of compounds, their whole biological activity is hard to explain. Therefore, research on the antioxidant activity of essential oils typically indicates that other minor chemical constituents that may interact synergistically or antagonistically to produce an additive and effective system against free radicals may also be responsible for the antioxidant activity [68,73].

### 3.5. Herbicidal (Phytotoxic) Activity

The tested samples demonstrated notable phytotoxic activity against seed germination and seedling growth of the wild radish (*R. raphanistrum*) in a concentration-dependent manner. At the highest concentration (100 µL/mL), VAO showed inhibition of seed germination, root growth, and shoot growth of *R. raphanistrum* by 66.67%, 96.66%, and 89.09%, respectively, VNO showed inhibition values of 90.0%, 89.39%, and 97.57%, respectively, while VTO showed inhibition values of 100%, 99.39%, and 92.12%, respectively (Table 2, Table 3 and Table 4). Based on the IC_50_ values, VAO showed IC_50_ values of 82.89, 19.468, and 37.95 µL/mL regrading seed germination, root growth, and shoot growth, respectively. For VNO, the IC_50_ values were 50.13, 47.06, and 16.75 µL/mL, respectively. For VTO, the IC_50_ values were 29.5, 9.33, and 27.13 µL/mL, respectively (Table 2, Table 3 and Table 4).

The phytotoxic potential of EOs from various *Vitex* species such as *V. agnus castus*, *V. negundo V. simplicifolia* has also been reported previously in other plants and weeds [15]. However, there is no study reported on phytotoxic potential of *V. trifolia.* Based on the present study, it was evident that VTO was more effective against *R. raphanistrum* than VNO and VAO. The suppressing effect of VTO on *R. raphanistrum* could be due to high amounts of β-caryophyllene (16.2%) and the synergetic effect of β-caryophyllene with other major and minor compounds present in the oil. In previous reports, β-caryophyllene was found to be responsible for the inhibition of germination and seedling growth of several plant species such as *Brassica campestris*, *Raphanus sativus*, *Vigna radiata*, and *Solanum lycopersicum* [22]. VNO also showed good inhibition values for seed germination and shoot growth, while VAO showed better inhibition value for root growth. The inhibition effect of samples could be due to the presence of phytotoxic compounds such as β-caryophyllene, 1,8-cineole, and sabinene, which are the main components in essential oil possessing phytotoxic activity [22,74]. In addition, 1,8-cineole was reported to interfere with the normal growth *Nicotiana tabacum* by blocking the DNA synthesis in their cell nuclei and organelles in root apical meristem cells [75]. Studies have also demonstrated that the terpenoids in EOs have a phytotoxic effects on plants, resulting in morphological and physiological alterations in the cells that impair plant growth [76].

### 3.6. Correlation of Essential Oil Components and Biological Activities

**Figure 5:**
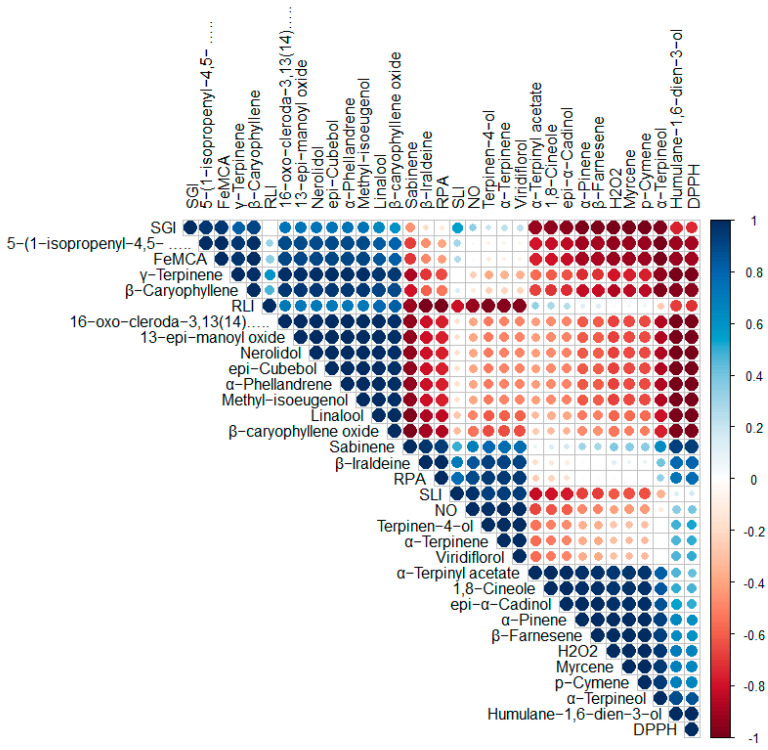
Correlation among chemical components of essential oils and biological activities of *Vitex* species (here, DPPH = percent inhibition of DPPH radical scavenging activity at 50 µL/mL; H_2_O_2_ = percent inhibition of H_2_O_2_ radical scavenging activity at 50 µL/mL; NO = percent inhibition of NO radical scavenging activity at 50 µL/mL; RPA = percent inhibition of reducing power activity at 50 µL/mL; FeMCA = percent inhibition of Fe^2+^ metal chelating activity at 50 µL/mL; SGI = percent inhibition of seed germination at 100 µL/mL; RLI = percent inhibition of root length at 100 µL/mL; SLI = percent inhibition of shoot length at 100 µL/mL.

Pearson’s correlation coefficient of major essential oil constituents (>2.00%) and antioxidant and herbicidal activities of *Vitex* species revealed that 5-(1-isopropenyl-4,5-dimethylbicyclo[4.3.0]nonan-5-yl)-3-methyl-2-pentenol acetate and β-caryophyllene strong positive correlation with DPPH radical scavenging activity and Fe^2+^ metal chelating activity, whereas γ-terpinene was also found positively correlated with Fe^2+^ metal chelating activity. Dahham et al. [77] also reported significant DPPH scavenging activity of β-caryophyllene. α-terpinyl acetate, 1,8-cineole and *epi*-α-cadinol, α-pinene, and β-farnesene showed moderate correlation with H_2_O_2_ radical scavenging activity. Terpinen-4-ol, α-terpinene, and viridiflorol showed moderate correlation with NO radical scavenging activity, as well as reducing power activity. The terpinen-4-ol was also reported to induce relaxation and was unlikely to be mediated by induction of NO release in rabbit duodenum relaxation in rabbit duodenum [78]. In terms of herbicidal activity of tested essential oils, 5-(1-isopropenyl-4,5-dimethylbicyclo[4.3.0]nonan-5-yl)-3-methyl-2-pentenol acetate and β-caryophyllene showed strong positive correlation with seed germination inhibition, whereas linalool and β-caryophyllene have moderated correlation with root length inhibition and terpinen-4-ol, α-terpinene, and viridiflorol were found to have strong positive correlation with shoot length inhibition. High β-caryophyllene-containing plants were also reported to have phytotoxic effects on weed species [79]. The correlation coefficient results were also supported by in vitro activities in presentation investigation, as well as previously reported studies. Pearson’s correlation coefficient representation of essential oil constituents with their biological activities is demonstrated in Figure 5.

### 3.7. Molecular Docking

**Figure 6:**
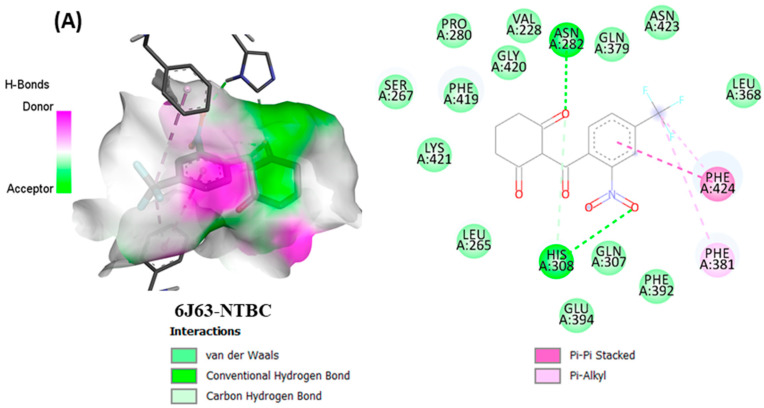

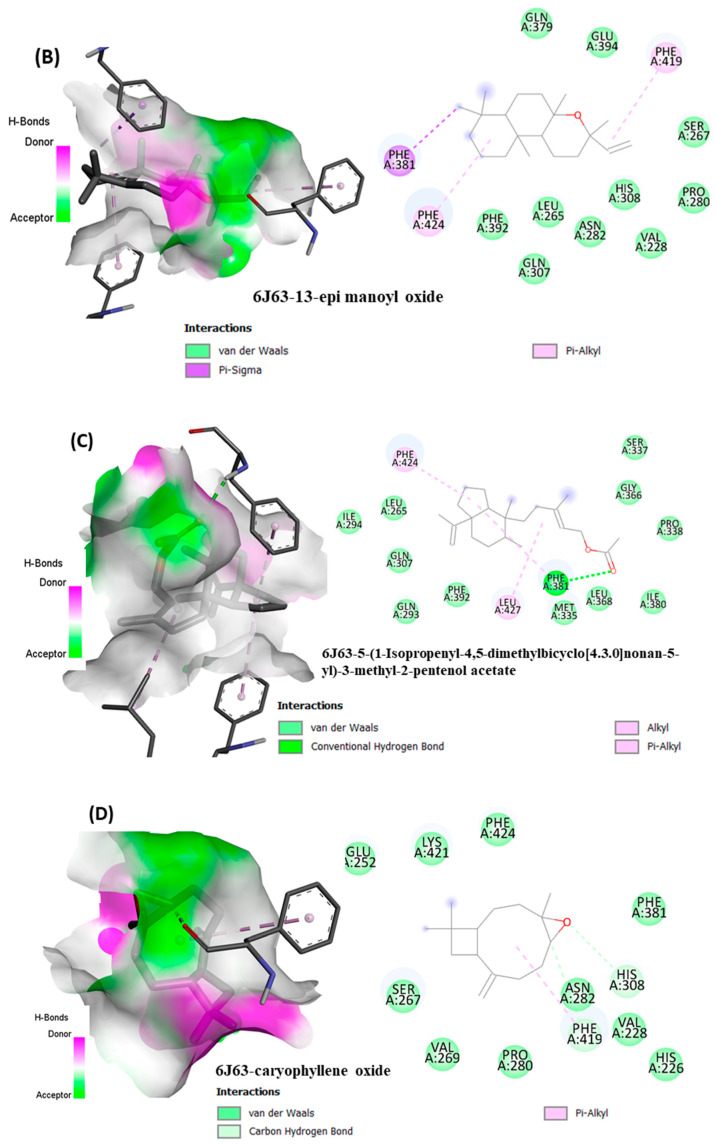

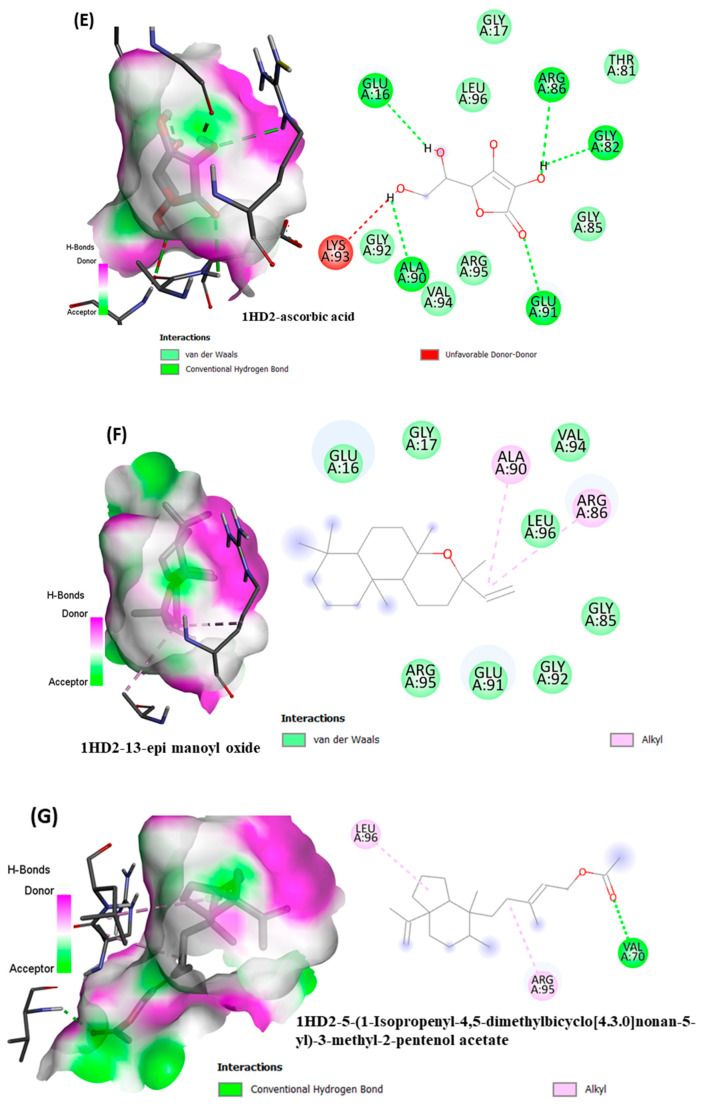

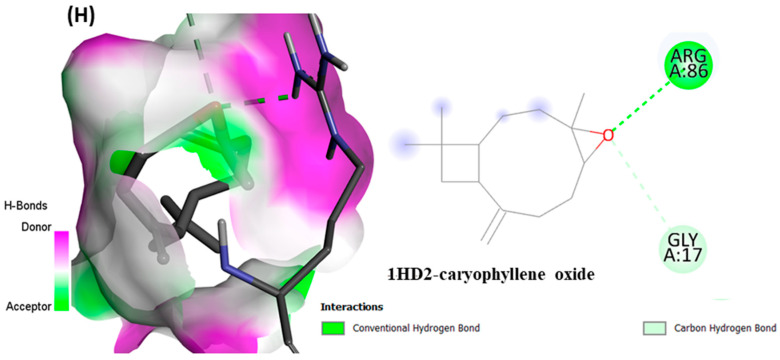
(**A**–**H**) Docked conformations of molecules in the binding cavity of HPPD (PDB: 6J63) and human periredoxin 5 (PDB: 1HD2) with least binding energies. The complex established are (**A**) 6J63-NTBC, (**B**) 6J63-13-*epi*-manoyl oxide, (**C**) 6J63-5-(1-Isopropenyl-4,5-dimethylbicyclo [4.3.0]nonan-5-yl)-3-methyl-2-pentenol acetate, (**D**) 6J63-caryophyllene oxide, (**E**) 1HD2-ascorbic acid; (**F**) 1HD2-13-*epi*-manoyl oxide, (**G**) 1HD2-5-(1-Isopropenyl-4,5-dimethylbicyclo [4.3.0]nonan-5-yl)-3-methyl-2-pentenol acetate, (**H**) 1HD2-caryophyllene oxide.

**Figure 7:**
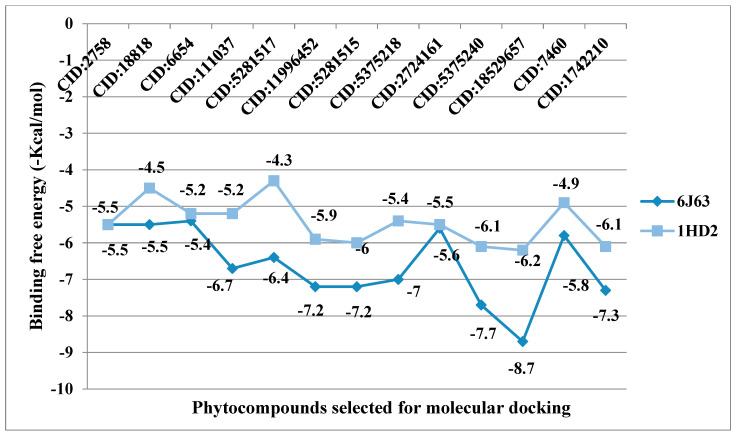
Binding energy (−kcal/mol) of selected phytocompounds from VAO, VNO, and VTO, complexed with 6J63 and 1HD2.

From in vitro studies, it was found that essential oils have potent antioxidant and phytotoxic activity. We also examined whether the major phytoconstituents from VAO, VNO, and VTO physically bind with antioxidant protein (human peroxiredoxin 5, PDB: 1HD2) and 4-hydroxyphenylpyruvate dioxygenase (HPPD, PDB: 6J63) receptors. The tested essential oils displayed good inhibition of the free radicals, for which the enzyme human peroxiredoxin 5 was selected, as it has broader activity against the reactive oxygen species (ROS) and is mostly involved in the stress protection mechanism [80,81]. The reason for selecting HPPD is that it is known to be the target protein for compounds with post-emergence herbicidal activity. In our results, the tested essential oils were found to have good post-emergence herbicidal activity against the receptor species, for which HPPD was selected as a target enzyme [18,38]. Among all selected phytocompounds, 13-*epi*-manoyl oxide demonstrated the best binding affinity with human peroxiredoxin 5 (−6.2 kcal mol) and HPPD (−8.7 kcal/mol). By introspecting the multiple dock poses, the best docked pose was selected as having the lowest binding energy. The best docked pose of 13-*epi*-manoyl oxide exhibited 2 pi-alkyl interaction, 1 pi-sigma interaction, and other Van der Waal interactions with 6J63 containing amino acid residues such as Phe A:424, Phe A:419, and Phe A:381, as represented in Figure 6B. Similarly, the best docked pose of 13-*epi*-manoyl oxide exhibited alkyl interaction with 1HD2 containing amino acid Ala A:90, Arg A:86, and exhibited Van der Waal interaction. For comparison purposes, a docking study of Nitisinone (CID:115355) was also performed with HPPD. Nitisinone (2-[2-nitro-4-(trifluoromethyl)benzoyl]cyclohexane-1,3-dione, (NTBC)) is a known inhibitor of HPPD. The docking study of ascorbic acid (CID:54670067), a known antioxidant, was performed with 1HD2. The binding energy for NTBC complexed with 6J63 was −8.9 kcal/mol, which is very close to that of 13-*epi*-manoyl oxide (−8.7 kcal/mol). On the other hand, binding energy of ascorbic acid complexed with 1HD2 came out to be −5.7 kcal/mol, which was higher than most of the compounds such as 13-*epi*-manoyl oxide (−6.2 kcal/mol), caryophyllene oxide (−6.1 kcal/mol), 5-(1-isopropenyl-4,5-dimethylbicyclo [4.3.0]nonan-5-yl)-3-methyl-2-pentenol acetate (−6.1 kcal/mol), β-caryophyllene (−6.0 kcal/mol), and viridiflorol (−5.9 kcal/mol), as shown in Figure 7. The lower values of binding free energy demonstrate more significant interaction between the receptor and the ligand. Our results were consistent with previous in silico studies reported by Alminderej et al. [73], where a phenylpropanoid-rich *Piper cubeba* EO gave similar results in terms of a proposed in vitro antioxidant activity by targeting human periredoxin 5. In this study, the compounds viridiflorol and caryophyllene oxide showed significant interaction with 1HD2 receptor as in the present study. In a recent study, focusing on the phytotoxic potential of *Calycolpus goetheanus* EO, it was found that the major components of the specimen, 1,8-cineole and β-caryophyllene interacted favorably with the HPPD protein [18]. These results are in general agreement with those obtained in the present study.

The listed binding energies of the volatiles docked with human peroxiredoxin 5 and HPPD (Figure 7) were found to be in the range −6.2 to −4.3 kcal/mol and −8.7 to −5.4 kcal/mol, respectively. Based on the study, it was observed that the major constituents interacted favorably with the receptors—most of which are the Van der Waal interactions. The analysis of ligand recognition reveals that the compounds can be good antioxidant and phytotoxic agents. Figure 6A–H shows the interaction of selected volatiles with the receptors (6J63 and 1HD2) having the least binding energies (higher docking scores), along with their 2D interaction with amino acid residues.

### 3.8. ADMET Analysis

The forecasting of ADME (absorption, distribution, metabolism, and excretion) properties of the selected compounds, including their pharmacokinetic and drug-like properties, have been estimated using SwissADME online server (http://www.swissadme.ch/, accessed on 12 August 2022). The collective laws of Lipinski’s [82], Egan’s [83], and Veber’s [84], which determine the properties of a drug, were followed. According to the rule that the compound should not violate more than 1 Lipinski rule, molecular weight (MW) < 500, topological surface area (TPSA) < 140, number of H-bond acceptors (nOHA) ≤ 5, number of H-bond donors (nOHD) ≤ 5, water partition coefficient (WLOGP) ≤ 5.88, number of rotatable bonds (nRB) ≤ 10. Based on the current findings, 12 out of 13 compounds selected followed the Lipinski’s, Egan’s, and Verber’s rule, indicating the good drug-like properties of the compounds. The bioavailability score was found to be 0.55 for all the compounds selected, indicating higher bioactivity of the molecule. The compounds share TPSA values less than 30 Å^2^, indicating good brain penetration and good lipophilicity behavior, with the consensus Log Po/w coming in the range 2.60–5.14 (Table 5). There was no P-glycoprotein (P-gp) substrate found, suggesting the good intestinal absorption of compounds. Except sabinene, α-pinene, β-farnesene, β-caryophyllene, 13-*epi*-manoyl acetate, and α-phellandrene, all compounds showed high gastrointestinal absorption. The compounds that were predicted to not cross the blood–brain barrier (BBB) were β-farnesene, β-caryophyllene, 5-(1-isopropenyl-4,5-dimethylbicyclo[4.3.0]nonan-5-yl)-3-methyl-2-pentenol acetate, and 13-*epi*-manoyl oxide.

**Figure 8:**
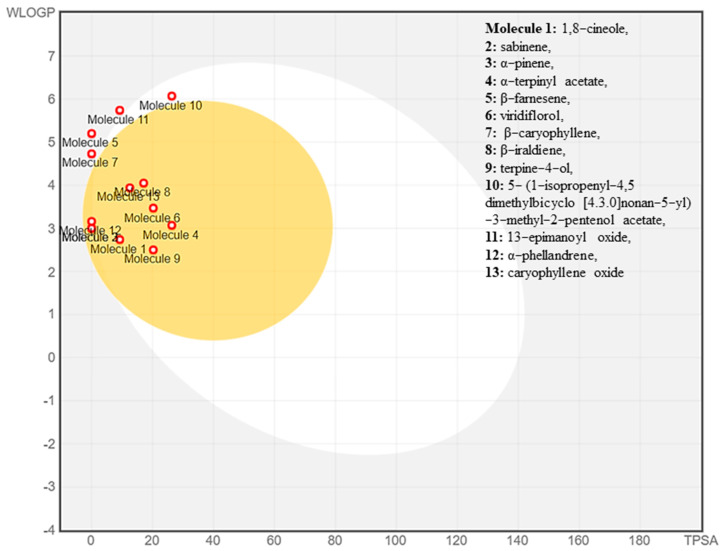
Boiled-egg graph of the selected phytoconstituents.

**Figure 9:**
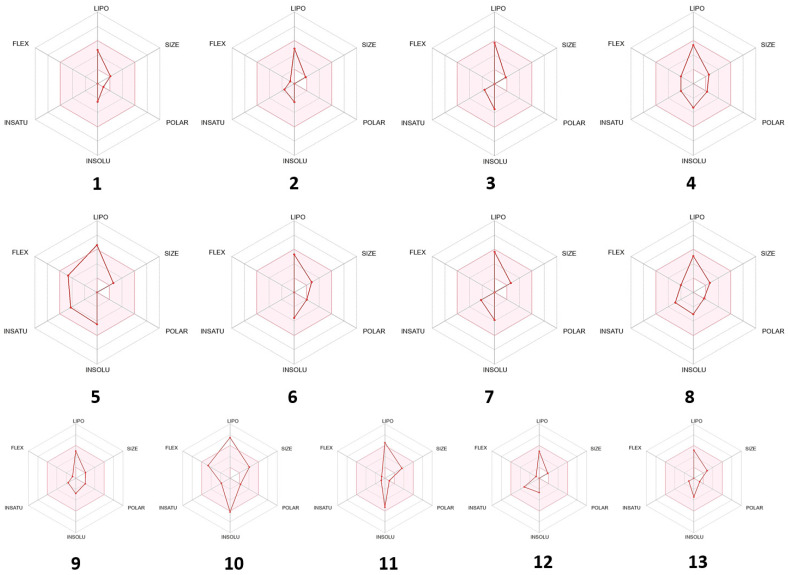
Bioavailability radar of selected phytoconstituents (pink area showed the drug likeness properties of selected compounds) 1: 1,8-cineole, 2: sabinene, 3: α-pinene, 4: α-terpinyl acetate, 5: β-farnesene, 6: viridiflorol, 7: β-caryophyllene, 8: β-iraldiene, 9: terpine-4-ol, 10: 5-(1-isopropenyl-4,5-dimethylbicyclo[4.3.0]nonan-5-yl)-3-methyl-2-pentenol acetate, 11: 13-epimanoyl oxide, 12: α-phellandrene, 13: caryophyllene oxide.

Some of the compounds interacted mainly with two isoenzymes of the cytochrome (CYP) family, namely CYP2C19 and CYP2C9, suggesting their efficiency while having minimal toxicity. Drug-like properties and GI absorption of selected compounds from VAO, VNO, and VTO were also represented by the boiled-egg prediction (Figure 8) and bioavailability radar graph (Figure 9). The compounds present in the yellow zone in the boiled-egg graph can permeate through the blood–brain barrier (BBB), and the pink area of the bioavailability radar graphs shows the drug-likeness of the compounds.

The toxicity parameters of selected phytocompounds were predicted using web server ProTox II (Table 6). All the selected compounds were predicted not to be hepatotoxic, carcinogenic, cytotoxic, immunotoxic, and mutagenic, except α-terpinyl acetate (hepatotoxic), 5-(1-isopropenyl-4,5-dimethylbic clo[4.3.0]nonan-5-yl)-3-methyl-2-pentenol acetate (carcinogenic), β-iraldiene, and caryophyllene oxide (immunotoxic). The LD_50_ values were also calculated to ensure the safety of the selected compounds as shown in Table 6. The compounds with LD_50_ > 2000 mg/kg, suggesting their safety for biological administration and as potential drugs.

## 4. Conclusions

In this study, the chemical diversity among the EOs obtained from three *Vitex* species from Tarai region, India, was revealed and analyzed. The chemical profile of EOs was characterized by high content of terpenoids. Moreover, the in vitro antioxidant and phytotoxic activities of the EOs were investigated to check the biological potentials of the plant-derived products of these *Vitex* species. All the tested EOs showed moderate to good antioxidant and phytotoxic potentials as assessed with different assays. The molecular docking study suggested that the compounds from the EOs can be good antioxidant and phytotoxic agents by the analysis of ligand interaction with the proteins. The ADMET analysis revealed the safety of most of the major compounds in the EOs. Overall, this study unveiled some interesting biological activities of these EOs, especially as natural antioxidants and phytotoxic agents, which justifies the use of the plant species in traditional medicine, as well as in the crop protection field. However, the in vivo study is necessary to investigate and assess the potency and safety of these EOs and their active components.

## Figures and Tables

**Table 1:** Comparative chemical composition of essential oil of *Vitex* species.

S. No.	Compound Name	Molecular Formula	R.I.	% Composition		
				VAO	VNO	VTO
1	α-Thujene (MH)	C_10_H_16_	930	1.4	0.2	0.1
2	α-Pinene (MH)	C_10_H_16_	939	8.2	2.6	1.6
3	Sabinene (MH)	C_10_H_16_	975	13.3	19.4	2.0
4	β-Pinene (MH)	C_10_H_16_	979	1.2	0.4	0.3
5	Oct-1-en-3-ol	C_8_H_16_O	979	-	0.5	4.0
6	Myrcene (MH)	C_10_H_16_	990	3.1	0.7	0.1
7	α-Phellandrene (MH)	C_10_H_16_	1002	-	-	4.2
8	α-Terpinene (MH)	C_10_H_16_	1017	-	2.3	-
9	β-Phellandrene (MH)	C_10_H_16_	1029	-	1.0	-
10	1,8-Cineole (OM)	C_10_H_18_O	1031	25.0	1.2	2.1
11	β-Ocimene (MH)	C_10_H_16_	1044	1.5	-	-
12	γ-Terpinene (MH)	C_10_H_16_	1059	0.3	0.8	3.3
13	Linalool oxide (OM)	C_10_H_18_O_2_	1086	-	0.4	-
14	α-Terpinolene (MH)	C_10_H_16_	1088	-	1.2	-
15	*p*-Cymene (MH)	C_10_H_12_	1091	2.2	0.6	0.2
16	Linalool (OM)	C_10_H_18_O	1096	0.8	0.6	2.2
17	*trans*-Sabinenehydrate (OM)	C_10_H_18_O	1098	0.2	0.3	-
18	Isoamyl isovalerate (Fatty Acid Ester)	C_10_H_20_O_2_	1103	-	0.1	-
19	*cis*-*p*-menth-2-en-1-ol (OM)	C_10_H_18_O	1121	0.2	0.2	0.1
20	δ-Terpineol (OM)	C_10_H_18_O	1166	0.5	-	-
21	Terpinen-4-ol (OM)	C_10_H_18_O	1177	1.9	5.4	1.8
22	Cryptone (OM)	C_9_H_14_O	1185	0.1	-	-
23	α-Terpineol (OM)	C_10_H_18_O	1188	2.5	1.4	0.3
24	Myrtenol (OM)	C_10_H_16_O	1195	-	0.1	-
25	*cis*-Piperitol (OM)	C_10_H_18_O	1196	-	0.1	-
26	γ-Terpineol (OM)	C_10_H_18_O	1199	0.1	-	-
27	*trans*-Piperitol (OM)	C_10_H_18_O	1208	-	-	0.8
28	β-Citronellol (OM)	C_10_H_20_O	1225	1.4	-	0.5
29	*cis*-Verbenyl acetate (OM)	C_12_H_18_O_2_	1282	1.1	-	-
30	Dihydroedulan II	C_13_H_22_O_2_	1284	0.1	0.1	0.5
31	Theaspirane A (OM)	C_13_H_22_O_2_	1290	-	1.5	0.2
32	α-Terpinyl acetate (OM)	C_12_H_20_O_2_	1349	5.5	-	0.5
33	β-Citronellyl acetate (OM)	C_12_H_22_O_2_	1352	1.3	-	-
34	β-Damascenone (OM)	C_13_H_18_O	1384	-	0.1	-
35	β-Bourbonene (SH)	C_15_H_24_	1388	-	-	0.7
36	β-Elemene (SH)	C_15_H_24_	1390	-	-	0.8
37	β-Caryophyllene (SH)	C_15_H_24_	1419	3.7	7.5	16.2
38	Methyl-isoeugenol (Phenylpropanoid)	C_11_H_14_O_2_	1453	-	-	3.1
39	α-Humulene (SH)	C_15_H_24_	1454	0.2	0.4	1.4
40	β-Farnesene (SH)	C_15_H_24_	1456	4.5	0.6	-
41	β-Selinene (SH)	C_15_H_24_	1490	-	0.3	-
42	*epi*-Cubebol (OS)	C_15_H_26_O	1494	-	-	2.3
43	Bicyclogermacrene (SH)	C_15_H_24_	1500	0.3	-	-
44	α-Muurolene (SH)	C_15_H_24_	1500	-	-	1.5
45	γ-Cadinene (SH)	C_15_H_24_	1513	-	-	0.8
46	δ-Cadinene (SH)	C_15_H_24_	1523	-	-	0.5
47	Hedycaryol (OS)	C_15_H_26_O	1548	-	0.5	-
48	β-Iraldeine (ionone)	C_14_H_22_O	1557	3.8	6.4	2.0
49	Nerolidol (OS)	C_15_H_26_O	1563	-	-	2.2
50	Spathulenol (OS)	C_15_H_24_O	1578	1.4	-	-
51	β-caryophyllene oxide (OS)	C_15_H_24_O	1583	1.9	1.3	4.6
52	Globulol (OS)	C_15_H_26_O	1590	-	0.2	1.4
53	Viridiflorol (OS)	C_15_H_26_O	1592	-	17.8	-
54	Ledol (OS)	C_15_H_26_O	1602	0.4	1.0	-
55	Humulene epoxide II (OS)	C_15_H_24_O	1608	-	-	1.2
56	Humulane-1,6-dien-3-ol (OS)	C_15_H_26_O	1619	2.4	2.3	-
57	*epi*-α-Cadinol (OS)	C_15_H_26_O	1640	2.1	-	-
58	α-Muurolol (OS)	C_15_H_26_O	1646	0.1	0.2	1.7
59	β-eudesmol (OS)	C_15_H_26_O	1650	0.1	1.1	-
60	Pogostol (OS)	C_15_H_26_O	1653	-	-	1.6
61	Drimenol (OS)	C_15_H_26_O	1767	0.3	0.4	0.8
62	Flourensadiol (OS)	C_15_H_26_O_2_	1870	0.1	-	-
63	Cubitene (DT)	C_20_H_32_	1878	-	1.3	-
64	Phytol (OD)	C_20_H_40_O	1943	-	1.2	1.3
65	13-epi-manoyl oxide (OD)	C_20_H3_4_O	2002	-	-	5.6
66	Manool (OD)	C_20_H3_4_O	2057	0.5	1.9	1.7
67	Sclareolide (OD)	C_16_H_26_O_2_	2066	-	0.2	-
68	Sclareol (OD)	C_20_H_36_O_2_	2223	-	1.7	1.0
69	5-(1-isopropenyl-4,5-dimethylbicyclo[4.3.0]nonan-5-yl)-3-methyl-2-pentenol acetate	C_22_H_36_O_2_	2265	-	5.2	11.7
70	Larixol (OD)	C_20_H_34_O_2_	2266	-	0.7	-
71	Verticiol (OD)	C_20_H_34_O	2273	-	-	1.4
72	16-oxo-cleroda-3,13(14)-(e)-dien-15-oic acid (OD)		-	-	-	2.8
Monoterpene hydrocarbon (MH)				31.2	29.4	11.8
Oxygenated monoterpene (OM)				40.6	11.3	8.5
Sesquiterpene hydrocarbon (SH)				8.7	8.8	21.9
Oxygenated sesquiterpene (OS)				8.8	24.8	15.8
Diterpene hydrocarbon (DT)				-	1.3	-
Oxygenated diterpene (OD)				0.5	5.7	13.8
Other than terpenoids				3.9	12.3	21.3
Total				93.7	93.6	93.1

VAO = *V. agnus-castus*; VNO = *V. negundo*; VTO = *V. trifolia*; RI value = Retention index value on a DB-5MS column in reference Adams, 2007 [22], or on NIST webbook [23]. MH = Monoterpene hydrocarbon; OM = Oxygenated monoterpene; SH = Sesquiterpene hydrocarbons; OS = Oxygenated sesquiterpene; DT = Diterpenene hydrocarbon; OD = Oxygenated diterpene.

**Table 2:** Mean % inhibition and IC_50_ values for seed germination inhibition by tested essential oils.

Samples	% Inhibition of Seed Germination					IC_50_ Values (µL/mL) in Triplicates			Mean IC_50_ Values (µL/mL) ± SD
	20 µL/mL	40 µL/mL	60 µL/mL	80 µL/mL	100 µL/mL	I	II	III	
VAO	3.33 ± 5.77 ^h^	3.33 ± 5.77 ^h^	40 ± 0.00 ^f^	43.33 ± 5.77 ^ef^	66.66 ± 5.77 ^cd^	78.94	81.17	88.57	82.89 ± 5.04
VNO	23.33 ± 5.77 ^g^	43.33 ± 5.77 ^ef^	56.66 ± 5.77 ^de^	76.66 ± 5.77 ^bc^	90.00 ± 0.00 ^ab^	51.11	55.294	44.00	50.13 ± 5.7
VTO	33.33 ± 5.77 ^fg^	60.00 ± 0.00 ^d^	90.00 ± 0.00 ^ab^	93.33 ± 5.77 ^a^	100.00 ± 0.00 ^a^	31.764	25.00	31.764	29.50 ± 3.9
Pendimethalin	100.00 ± 0.00	100.00 ± 0.00	100.00 ± 0.00	100.00 ± 0.00	100.00 ± 0.00				

VAO = *V. agnus-castus*; VNO = *V. negundo*; VTO = *V. trifolia*; SD = standard deviation; According to Tukey’s test (*p* < 0.05), mean values that are followed by the same letter inside a column are not statistically different from one another.

**Table 3:** Mean % inhibition and IC_50_ values for root length inhibition by tested essential oils.

Samples	% Inhibition of Root Length					IC_50_ Values (µL/mL) in Triplicates			Mean IC_50_ Values (µL/mL) ± SD
	20 µL/mL	40 µL/mL	60 µL/mL	80 µL/mL	100 µL/mL	I	II	III	
VAO	45.15 ± 1.0 ^g^	66.36 ± 0.9 ^d^	80.90 ± 0.9 ^c^	89.69 ± 0.5 ^b^	96.66 ± 0.5 ^a^	18.963	19.161	20.28	19.468 ± 0.7
VNO	32.27 ± 1.6 ^h^	45.45 ± 0.9 ^g^	58.48 ± 1.0 ^e^	68.78 ± 2.2 ^d^	89.39 ± 2.6 ^b^	46.529	47.00	47.67	47.06 ± 0.5
VTO	52.42 ± 2.2 ^f^	70.00 ± 1.8 ^d^	83.03 ± 1.3 ^c^	92.12 ± 1.04 ^b^	99.39 ± 1.04 ^a^	9.766	9.766	8.479	9.337 ± 0.7
Pendimethalin	100.00 ± 0.00	100.00 ± 0.00	100.00 ± 0.00	100.00 ± 0.00	100.00 ± 0.00				

VAO = *V. agnus-castus*; VNO = *V. negundo*; VTO = *V. trifolia*; SD = standard deviation. According to Tukey’s test (*p* < 0.05), mean values that are followed by the same letter inside a column are not statistically different from one another.

**Table 4:** Mean % inhibition and IC_50_ values for shoot length inhibition by tested essential oils.

Samples	% Inhibition of Shoot Length					IC_50_ Values (µL/mL) in Triplicates			Mean IC_50_ Values (µL/mL) ± SD
	20 µL/mL	40 µL/mL	60 µL/mL	80 µL/mL	100 µL/mL	I	II	III	
VAO	37.57 ± 0.5 ^k^	52.72 ± 1.8 ^i^	63.48 ± 0.6 ^g^	77.57 ± 0.5 ^e^	89.09 ± 0.9 ^bc^	37.52	37.58	38.75	37.95 ± 0.6
VNO	51.21 ± 1.3 ^i^	64.24 ± 2.7 ^g^	74.54 ± 0.9 ^e^	83.78 ± 0.2 ^d^	97.57 ± 0.5 ^a^	17.43	17.42	15.41	16.75 ± 1.2
VTO	45.15 ± 0.5 ^j^	58.48 ± 1.0 ^h^	68.03 ± 0.6 ^f^	85.90 ± 1.2 ^cd^	92.12 ± 0.5 ^b^	28.65	26.417	26.33	27.13 ± 1.3
Pendimethalin	100.00 ± 0.01	100.00 ± 0.01	100.00 ± 0.0	100.00 ± 0.00	100.00 ± 0.00				

VAO = *V. agnus-castus*; VNO = *V. negundo*; VTO = *V. trifolia*; SD = standard deviation. According to Tukey’s test (*p* < 0.05), mean values that are followed by the same letter inside a column are not statistically different from one another.

**Table 5:** In silico ADMET analysis of major constituents of VAO, VNO, and VTO.

Entry	1	2	3	4	5	6	7	8	9	10	11	12	13
TPSA * (Å^2^)	9.23	0.00	0.00	26.30	0.00	20.23	0.00	17.07	20.23	26.30	9.23	0.00	12.53
Consensus * Log Po/w	2.67	3.25	3.44	3.04	4.97	3.42	4.24	3.56	2.60	4.25	5.14	2.97	3.68
Mol wt (g/mol)	154.25	136.23	136.23	196.29	204.35	222.37	204.35	206.32	154.25	332.52	290.48	136.23	220.35
nRB	0	1	0	3	7	0	0	3	1	7	1	1	0
nOHA	1	0	0	2	0	1	0	1	1	2	1	0	1
nOND	0	0	0	0	0	1	0	0	1	0	0	0	0
WLOGP	2.74	3.00	3.00	3.07	5.20	3.47	4.73	4.05	3.50	6.07	5.74	3.16	3.94
Water solubility	Soluble	Soluble	Soluble	Soluble	Soluble	Soluble	Soluble	Soluble	Soluble	Moderately	Moderately	Soluble	Soluble
GI absorption **	High	Low	Low	High	Low	High	Low	High	High	High	Low	Low	High
BBB permeant **	Yes	Yes	Yes	Yes	No	Yes	No	Yes	Yes	No	No	Yes	Yes
P-gp substrate **	No	No	No	No	No	No	No	No	No	No	No	No	No
CYP1A2 inhibitor **	No	No	No	No	Yes	No	No	No	No	No	No	No	No
CYP2C19 inhibitor **	No	No	No	No	No	Yes	Yes	No	No	Yes	Yes	No	Yes
CYP2C9 inhibitor **	No	No	Yes	Yes	Yes	No	Yes	No	No	Yes	Yes	No	Yes
CYP2D6 inhibitor **	No	No	No	No	No	No	No	No	No	No	No	No	No
CYP3A4 inhibitor **	No	No	No	No	No	No	No	No	No	No	No	No	No
Log *K_p_* (cm/s) (Skin permeation)	−5.30	−4.94	−3.95	−4.69	−3.27	−5.00	−4.44	−5.16	−4.93	−2.97	−3.86	−4.85	−5.12
Lipinski ***	Yes	Yes	Yes	Yes	Yes	Yes	Yes	Yes	Yes	Yes	Yes	Yes	Yes
Lipinski violation	0	1	1	0	1	0	1	0	0	1	1	0	0
Bioavailability score ***	0.55	0.55	0.55	0.55	0.55	0.55	0.55	0.55	0.55	0.55	0.55	0.55	0.55

VAO: *V. agnus-castus* essential oil, VNO: *V. negundo* essential oil, VTO: *V. trifolia* essential oil, ADMET: absorption, distribution, metabolism, excretion and toxicity, Lipophilicity *, Pharmacokinetics **, Drug Likeliness ***, TPSA: topological polar surface area, nRB: no. of rotable bonds, nOHA: no. of H-bond acceptor, nOHD: no. of H-bond donor, WLOGP: water partition coefficient, GI absorption: gastrointestinal absorption, BBB: blood–brain barrier, P-gp: permeability glycoprotein, CYP: cytochrome P450, Entry 1: 1,8-cineole, 2: sabinene, 3: α-pinene, 4: α-terpinyl acetate, 5: β-farnesene, 6: viridiflorol, 7: β-caryophyllene, 8: β-iraldiene, 9: terpine-4-ol, 10: 5-(1-isopropenyl-4,5-dimethylbicyclo [4.3.0]nonan-5-yl)-3-methyl-2-pentenol acetate, 11: 13-epimanoyl oxide, 12: α-phellandrene, 13: caryophyllene oxide.

**Table 6:** Toxicological properties of selected compounds from VAO, VNO, and VTO.

Compounds	Hepatotoxicity		Carcinogenicity		Cytotoxicity		Immunotoxicity		Mutagenicity		Predicted LD_50_ (mg/kg)	Toxicity Class
	Pr	Pb	Pr	Pb	Pr	Pb	Pr	Pb	Pr	Pb		
1,8-Cineole	NH	0.86	NC	0.68	NCy	0.75	NI	0.99	NM	0.96	2480	V
Sabinene	NH	0.81	NC	0.59	NCy	0.71	NI	0.51	NM	0.82	5000	V
α-Pinene	NH	0.86	NC	0.60	NCy	0.75	NI	0.99	NM	0.93	3700	V
α-Terpinyl acetate	H	0.53	NC	0.66	NCy	0.80	NI	0.97	NM	0.94	4800	V
β-Farnesene	NH	0.79	NC	0.73	NCy	0.81	NI	0.99	NM	0.98	5000	V
Viridiflorol	NH	0.77	NC	0.69	NCy	0.89	NI	0.87	NM	0.75	2000	IV
β-Caryophyllene	NH	0.80	NC	0.70	NCy	0.75	I	0.54	NM	0.95	5300	V
β-Iraldiene	NH	0.68	NC	0.79	NCy	0.78	NI	0.97	NM	0.93	4590	V
Terpine-4-ol	NH	0.80	NC	0.72	NCy	0.88	NI	0.99	NM	0.83	1016	IV
5-(1-isopropenyl-4,5-dimethylbicyclo[4.3.0]nonan-5-yl)-3-methyl-2-pentenol acetate	NH	0.68	C	0.58	NCy	0.76	NI	0.89	NM	0.87	5000	V
13-epi manoyl oxide	NH	0.86	NC	0.69	NCy	0.75	NI	0.71	NM	0.91	4300	V
α-Phellandrene	NH	0.83	NC	0.52	NCy	0.80	NI	0.88	NM	0.92	5700	VI
Caryophyllene oxide	NH	0.80	NC	0.57	NCy	0.79	I	0.83	NM	0.88	5000	V

Pr: Prediction, Pb: Probability, NH: Nonhepatotoxic, NC: Noncarcinogenic, NCy: Noncytotoxic, NI: Nonimmunotoxic, NM: Nonmutagenic, H: Hepatotoxic, I: Immunotoxic, C: Carcinogenic, Toxicity class: (Class I: fatal if swallowed (LD_50_  ≤  5), Class II: fatal if swallowed (5  <  LD_50_  ≤  50), Class III: toxic if swallowed (50  <  LD_50_  ≤  300), Class IV: harmful if swallowed (300  <  LD_50_  ≤  2000), Class V: may be harmful if swallowed (2000  <  LD_50_  ≤  5000), Class VI: non-toxic (LD_50_  >  5000)).

## Data Availability

Data are contained within the article.

## Supplementary Materials

The following supporting information can be downloaded at: https://www.mdpi.com/article/10.3390/antiox11101911/s1, Supplementary Material S1, ion-chromatograms, and mass spectra of the major compounds can be observed.
