# Supplementary material for: Phytochemical Profile, In Vitro Bioactivity Evaluation, In Silico Molecular Docking and ADMET Study of Essential Oils of Three Vitex Species Grown in Tarai Region of Uttarakhand
Source: Antioxidants (Basel). 2022 Sep 27;11(10):1911. doi: 10.3390/antiox11101911 (PMC9598352; doi:10.3390/antiox11101911)
Supplement: Supplementary file 1 [file antioxidants-11-01911-s001.zip › antioxidants-1915160-supplementary.pdf]

Supplementary material S1

Article

Phytochemical Profile, In Vitro Bioactivity Evaluation, In Silico Molecular Docking and ADMET Study of Essential Oils of Three *Vitex* Species Grown in Tarai Region of Uttarakhand

Himani Karakoti [1], Sonu Kumar Mahawer [1], Monika Tewari [1], Ravendra Kumar [1,*], Om Prakash [1], Mozaniel Santana de Oliveira [2,*] and Dharmendra Singh Rawat [3]

[1] Department of Chemistry, College of Basic Sciences and Humanities, G.B. Pant University of Agriculture and Technology, Pantnagar 263145, U.S. Nagar, Uttarakhand, India

[2] Campus de Pesquisa-Museu Paraense Emílio Goeldi-Botany Coordination, Av. Perimetral, 1901-Terra Firme, Belém 66077-830, PA, Brazil

[3] Department of Biological Sciences, College of Basic Sciences and Humanities, G.B. Pant University of Agriculture and Technology, Pantnagar 263145, U.S. Nagar, Uttarakhand, India

* Correspondence: ravendra.kumar@gbpuat-cbsh.ac.in (R.K.); mozanieloliveira@museu-goeldi.br (M.S.d.O.)

Abstract: A comparative study of volatiles, antioxidant activity, phytotoxic activity, as well as in silico molecular docking and ADMET study, was conducted for essential oils from three *Vitex* species, viz., *V. agnus-castus*, *V. negundo*, and *V. trifolia*. Essential oils (OEs) extracted by hydrodistillation were subjected to compositional analysis using GC-MS. A total number of 37, 45, and 43 components were identified in *V. agnus-castus*, *V. negundo*, and *V. trifolia*, respectively. The antioxidant activity of EOs, assessed using different radical-scavenging (DPPH, H_2O_2 and NO), reducing power, and metal chelating assays, were found to be significant as compared with those of the standards. The phytotoxic potential of the EOs was performed in the receptor species *Raphanus raphanistrum* (wild radish) and the EOs showed different levels of intensity of seed germination inhibition and root and shoot length inhibition. The molecular docking study was conducted to screen the antioxidant and phytotoxic activity of the major and potent compounds against human protein target, peroxiredoxin 5, and 4-hydroxyphenylpyruvate dioxygenase protein (HPPD). Results showed good binding affinities and attributed the strongest inhibitory activity to 13-*epi*-manoyl oxide for both the target proteins.

Keywords: natural products; bioactive compounds; antioxidant; phytotoxic; molecular modeling; virtual ligand screening

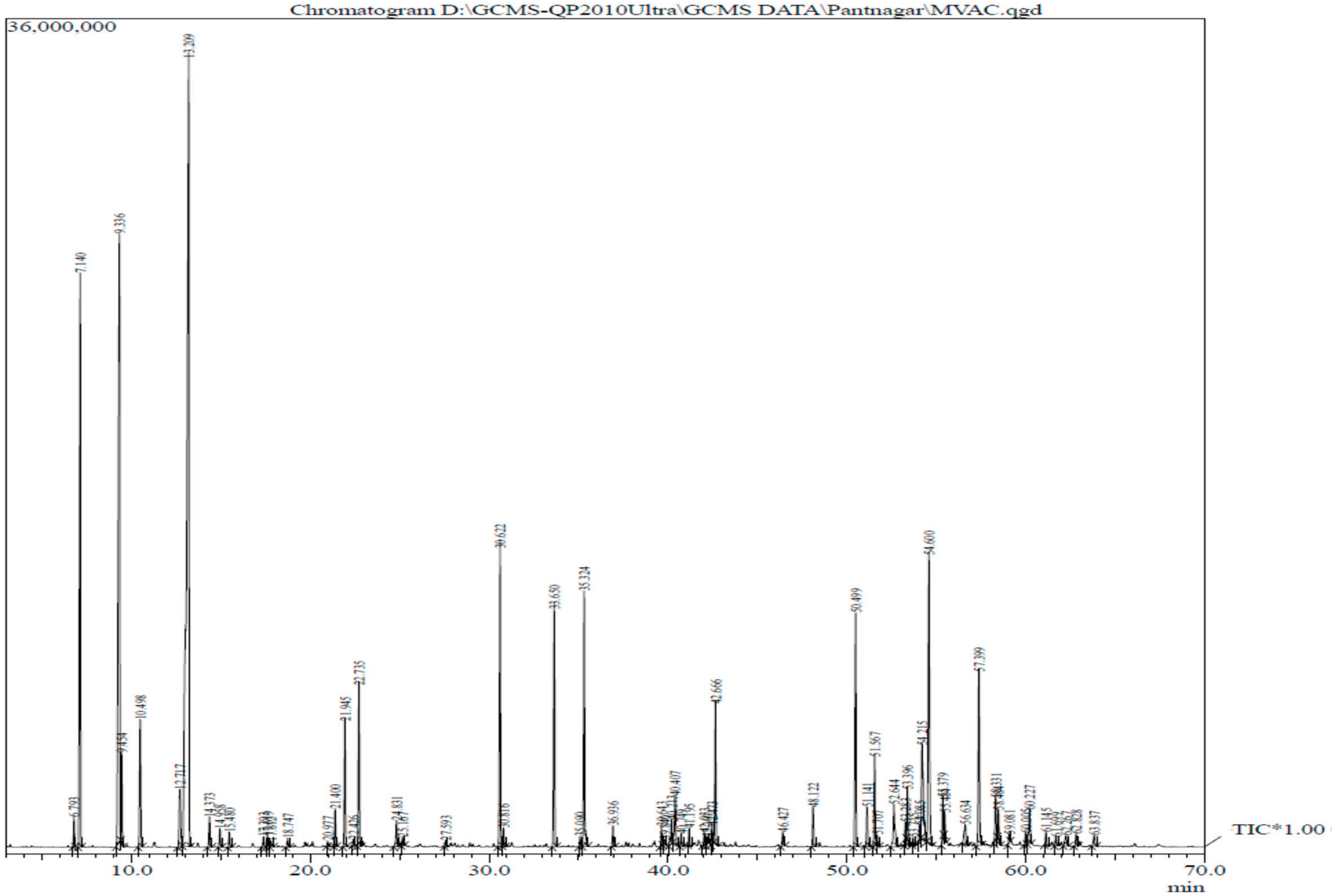

Figure S1. Ion-chromatogram of *Vitex agnus-castus* essential oil

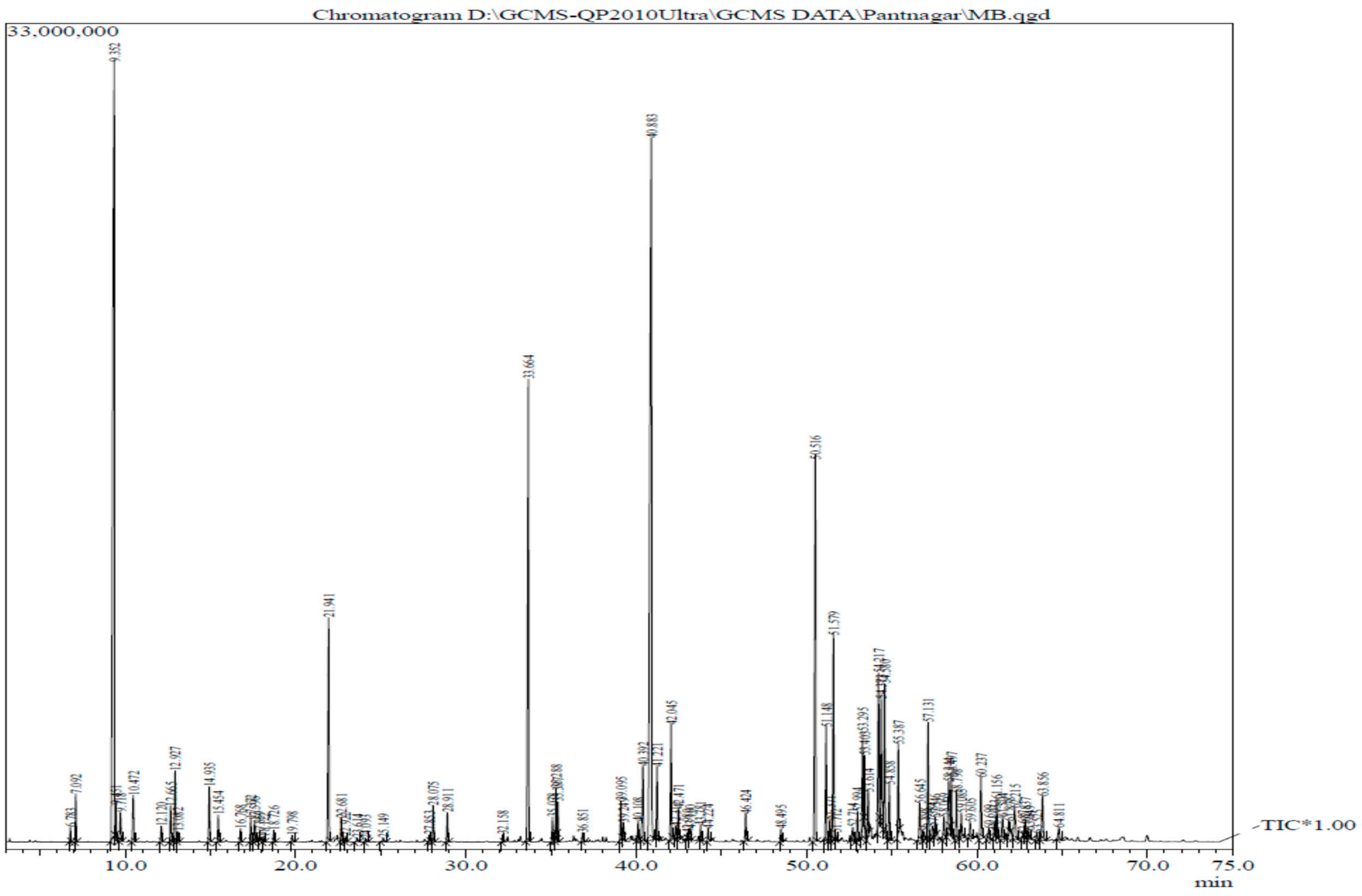

Figure S2. Ion-chromatogram of *Vitex negundo* essential oil

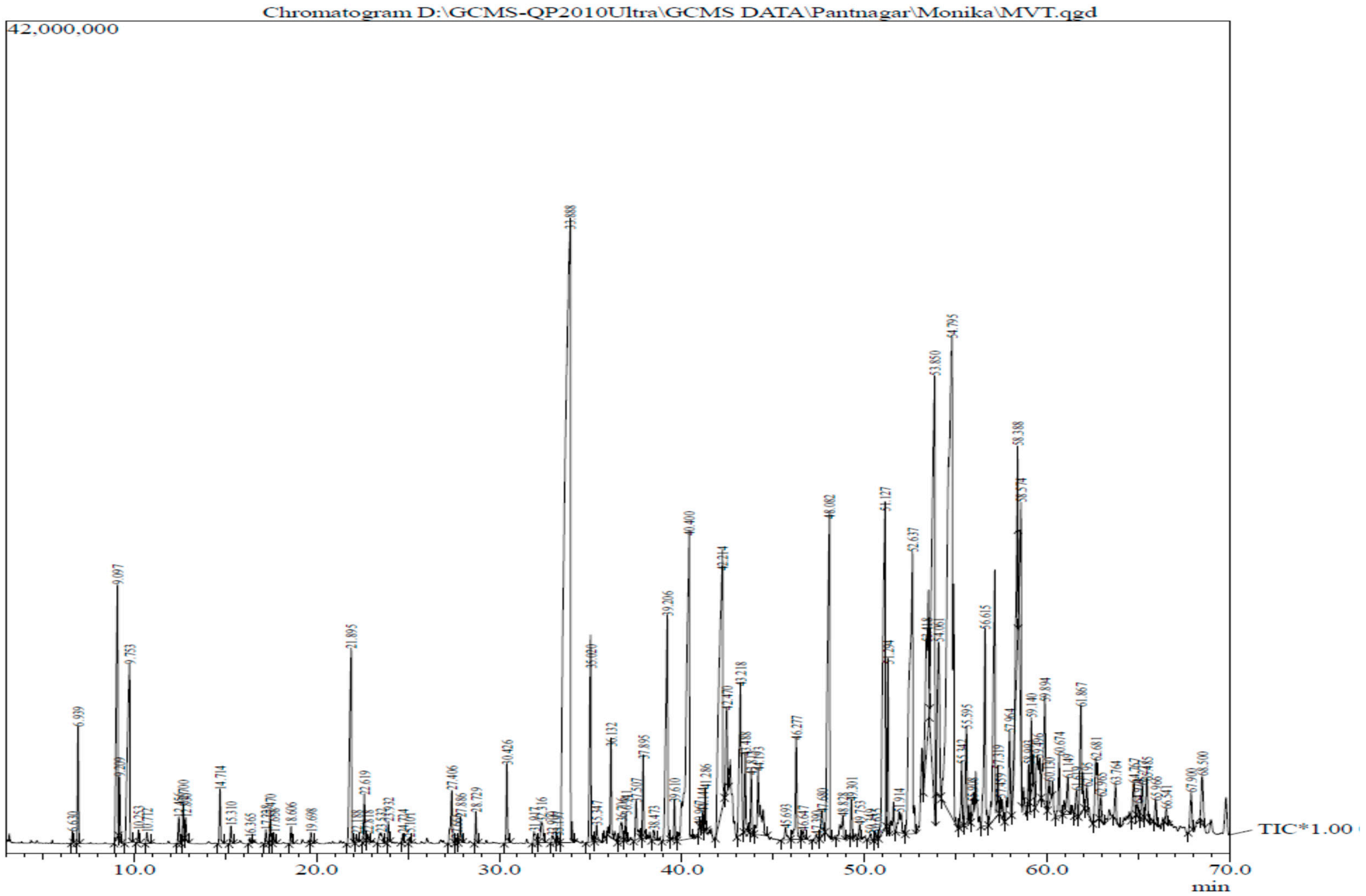

Figure S3. Ion-chromatogram of *Vitex trifolia* essential oil

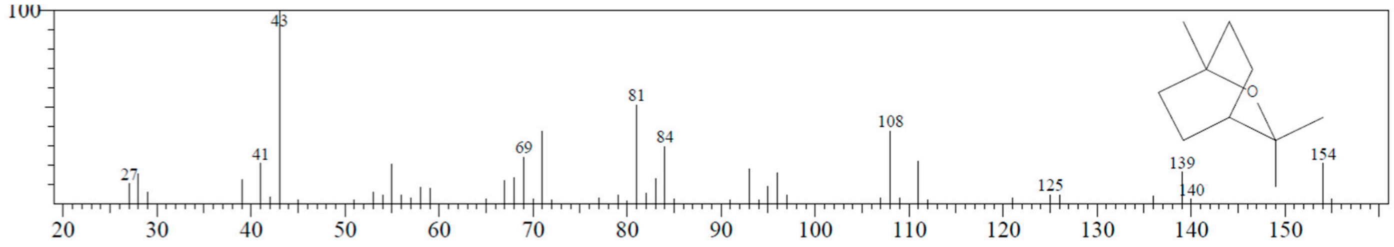

1,8-cineole

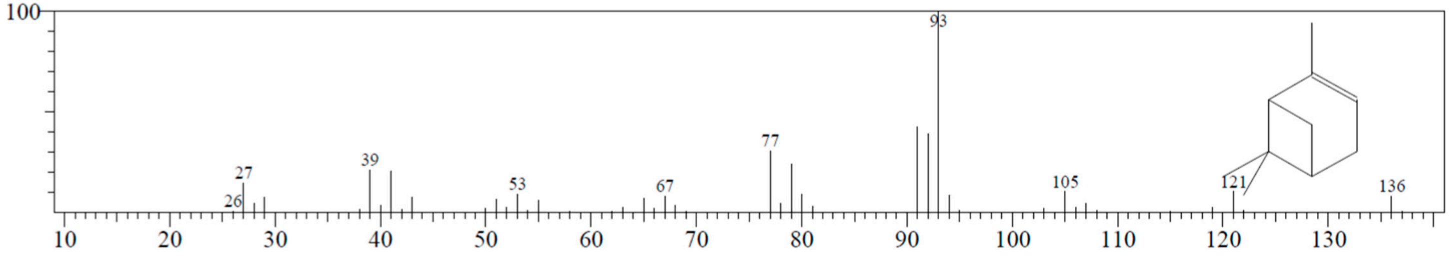

***α*-pinene**

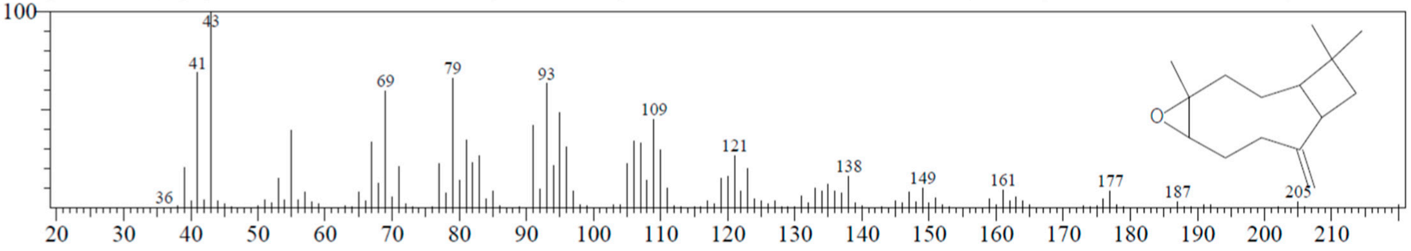

caryophyllene oxide

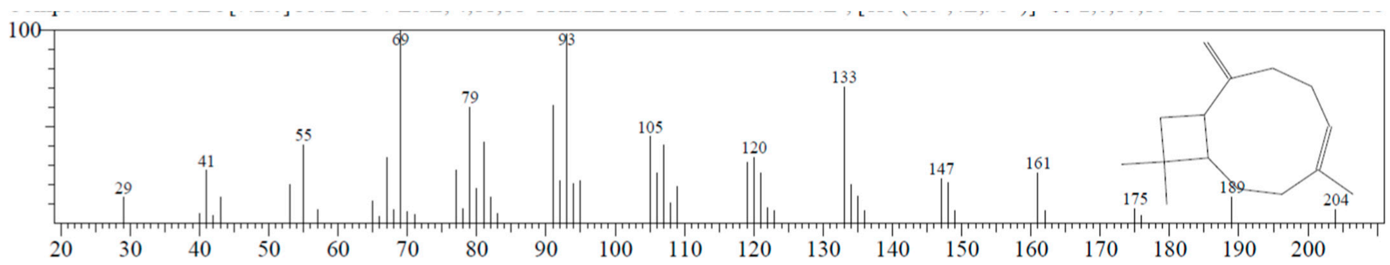

β-caryophyllene

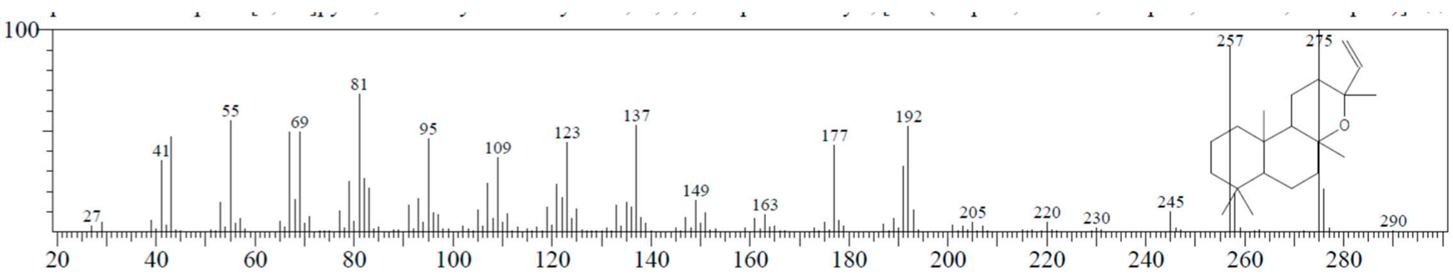

13-*epi*-manoyl oxide

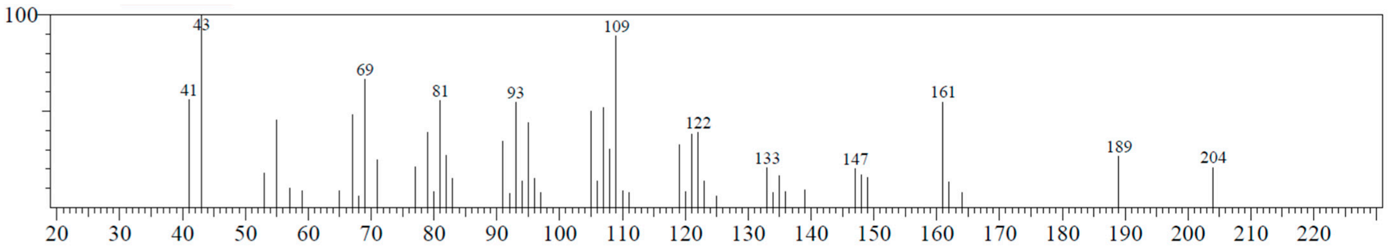

viridiflorol

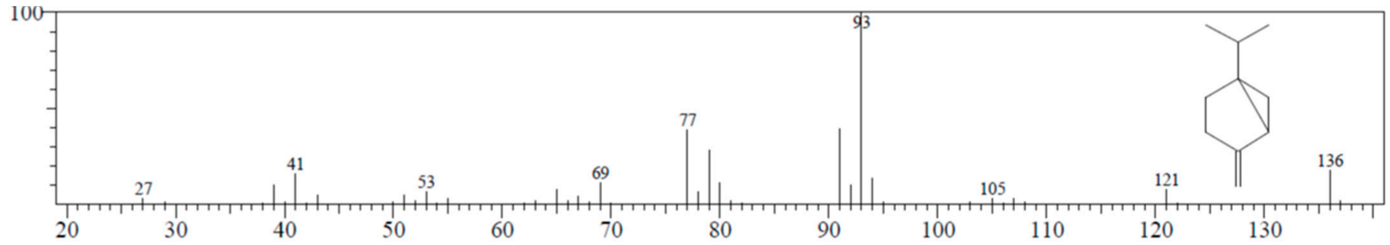

sabinene

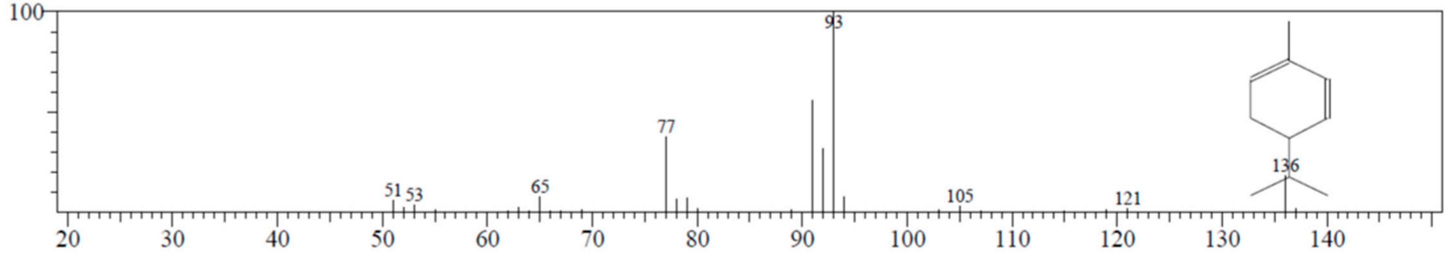

***α*-phellandrene**

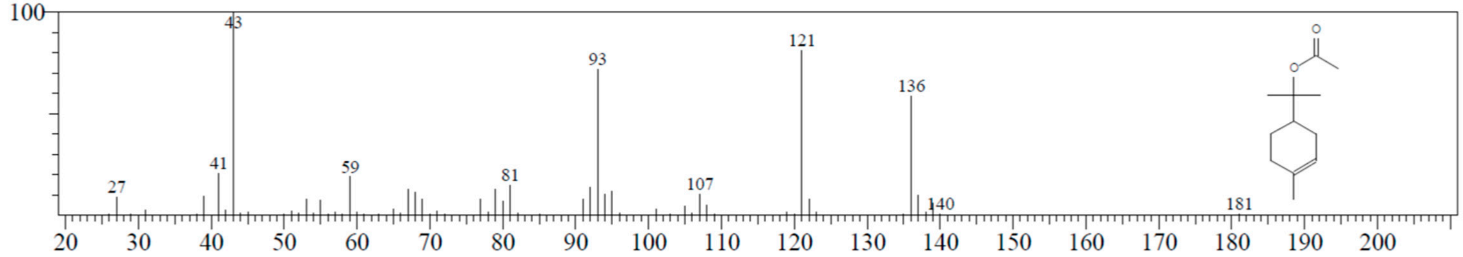

***α*-terpinyl acetate**

Mass spectra of some major compounds
